# Voltammetry at Hexamethyl-P-Terphenyl Poly(Benzimidazolium) (HMT-PMBI)-Coated Glassy Carbon Electrodes: Charge Transport Properties and Detection of Uric and Ascorbic Acid

**DOI:** 10.3390/s20020443

**Published:** 2020-01-13

**Authors:** Matthew Rees, Andrew G. Wright, Steven Holdcroft, Paolo Bertoncello

**Affiliations:** 1 Systems and Process Engineering Centre, College of Engineering, Swansea University, Bay Campus, Crymlyn Burrows, Swansea SA1 8EN, UK; 714909@swansea.ac.uk; 2 Department of Chemistry, Simon Fraser University, Burnaby, BC V5A 1S6, Canada; andrewgordonwright@gmail.com (A.G.W.); holdcrof@sfu.ca (S.H.); 3 Centre for NanoHealth, Swansea University, Singleton Campus, Swansea SA2 8PP, UK

**Keywords:** chemically modified electrodes, voltammetry, sensors, uric acid, ascorbic acid, hexamethyl-p-terphenyl poly(benzimidazolium), anion exchange polymer

## Abstract

We describe the voltammetric behavior of an anion-exchange membrane, hexamethyl-p-terphenyl poly(benzimidazolium) (HMT-PMBI). The anion-exchange properties of HMT-PMBI chemically modified electrodes were investigated using K_4_Fe(CN)_6_ and K_2_IrCl_6_ as redox probes. The permselectivity properties of HMT-PMBI chemically modified electrodes were ascertained using tris(2-2’)bipyridyl-ruthenium(II) chloride Ru(bpy)_3_^2+^. Cyclic voltammetry and chronoamperometry were utilized to extract parameters such as the concentration of the redox mediators inside the films and the apparent diffusion coefficients. We found the concentration of K_4_Fe(CN)_6_ and K_2_IrCl_6_ redox species within HMT-PMBI-coated films to be on the order of 0.04–0.1 mol·dm^−3^, and values of *D_app_* ca. 10^−10^–10^−9^ cm^2^·s^−1^. To evaluate the possibility of using such a polymer coating in electroanalysis, HMT-PMBI-modified electrodes were utilized for the voltammetric detection of uric acid in artificial urine, Surine^®^ and ascorbic acid in Vitamin C samples. The results showed that HMT-PMBI-coated electrodes can detect uric acid in Surine^®^ with a limit of detection (LoD) of 7.7 µM, sensitivity of 0.14 µA·µM^−1^·cm^−2^, and linear range between 5 μM and 200 μM, whereas for Vitamin C tablets, the LoD is 41.4 µM, the sensitivity is 0.08 µA·µM^−1^·cm^−2^, and the linear range is between 25 μM and 450 μM.

## 1. Introduction

There is ongoing interest in analytical chemistry in developing sensors with high sensitivity and selectivity to detect analytes of clinical interest at low concentrations while minimizing potential interferences. In this respect, amperometric sensors are very attractive compared to other analytical methods because of their low cost and the possibility of performing measurements in situ using portable devices [1,2,3]. One of the possibilities to obtain the desired sensitivity and selectivity is the modification of electrode surfaces with suitable molecules, so that they will be able to interact and preferentially preconcentrate the analyte of interest. This has led to the development of chemically modified electrodes, whose applications have been widely reported in the literature since the 1980s [2,3,4,5,6]. Ion-exchange voltammetry (IEC) is one of the techniques utilized to preconcentrate analytes at electrode surfaces [7], and as a result, ion-exchange polymers or ionomers have been extensively used for the modification of electrode surfaces. Originally, ionomers were synthesized for fuel cell applications, and specifically used as membranes to separate the anode from the cathode compartments, due to their very good transport of opposite charge ions [8,9,10,11]. For instance, in proton exchange fuel cells (PEMFCs), the ionomer is made of a polymer having a net negative charge (typically sulfonic or carboxylic) that transports protons from the anode to the cathode, whereas in alkaline fuel cells (AFCs), the ionomer is made of a polymer with a net positive charge (typically ammonium or phosphonium) that transports hydroxide ions from the cathode to anode. These ion-exchange properties made ionomers suitable as membranes in water electrolyzers and desalination units [12,13,14]. From an electroanalytical point of view, ionomer-coated electrodes are particularly attractive due to their very good ion-exchange properties, which allow the preconcentration of analytes having opposite charge even at very low concentrations, at the same time providing protection against potential interferences and fouling. An example is Nafion^®^, a cation exchange ionomer widely used for the determination of positively charged dopamine up to ultra-trace concentrations, whilst protecting the electrode from the potential interferences of ascorbic acid [15,16]. Instead, in the case of anion-exchange ionomers, the literature extensively reported ammonium functionalized ionomers [17,18,19,20,21]; more recently, we reported phosphonium-based ionomers [22,23]. These ionomers have also been extensively studied due to their charge transport mechanisms with various redox mediators [7,24,25,26,27,28,29,30,31,32]. Ionenes are polymers that contain an ionic amine in the backbone [33,34] and have shown great stability in alkaline fuel cells [35,36,37]. In this work, we studied for the first time the charge transport properties of a novel ionene derivative, namely hexamethyl-p-terphenyl poly(benzimidazolium) (HMT-PMBI), and evaluated its use as a chemically modified electrode for the detection of uric and ascorbic acid. First, we deposited a film of HMT-PMBI on glassy carbon electrodes and evaluated the charge transport properties with respect to standard negatively charged redox mediators such as K_4_Fe(CN)_6_ and K_2_IrCl_6_. The electrochemical characterization allowed the estimation of the concentration of the redox species inside the HMT-PMBI coatings and the apparent diffusion coefficients. Finally, we demonstrated the potentiality of HMT-PMBI-coated electrodes for the detection of uric acid in artificial urine, Surine^®^ and ascorbic acid in commercial Vitamin C tablets. Uric acid, along with oxypurine, is one of the products derived from purine derivatives as a result of human metabolism [38,39,40]. Typically, the concentration of uric acid in a healthy human being is in the millimolar range (180‒370 micromolar) in urine [41]; in the blood, the standard concentration is 120‒450 micromolar [42,43,44]. The presence of elevated concentrations of uric acid is associated with several diseases such as hyperuricemia [41,45,46,47], gout derived from excessive alcohol consumption [48], diabetes [49,50,51], obesity [52,53] and high cholesterol [54]. On the other hand, ascorbic acid (or Vitamin C) is well known for its reducing properties and is involved in many physiological processes such as scavenging for reactive oxygen species [55,56]. As such, Vitamin C is widely used as a supplement in the food industry [56]. Hence, the detection of uric and ascorbic acid is of relevance to analytical applications.

## 2. Materials and Methods

### 2.1. Materials

Potassium hexachloroiridate(IV), K_2_IrCl_6_, Potassium ferrocyanide K_4_Fe(CN)_6_, Tris(2-2’)bipyridyl-ruthenium(II) chloride, [Ru(bpy)_3_]^2+^, NaCl, uric acid, ascorbic acid and all other reagents were purchased from Sigma Aldrich (Dorset, UK) and used as received. Glassy carbon electrodes (GCEs) and materials used in polishing were obtained from IJ Cambria (UK). Aqueous solutions were made using Milli-Q Ultra-pure water (with resistivity of 18.2 MΩ cm at 25 °C) from Millipore (Watford, UK) Direct Q3 water dispenser.

### 2.2. Synthesis of HMT-PMBI

**Scheme 1:**
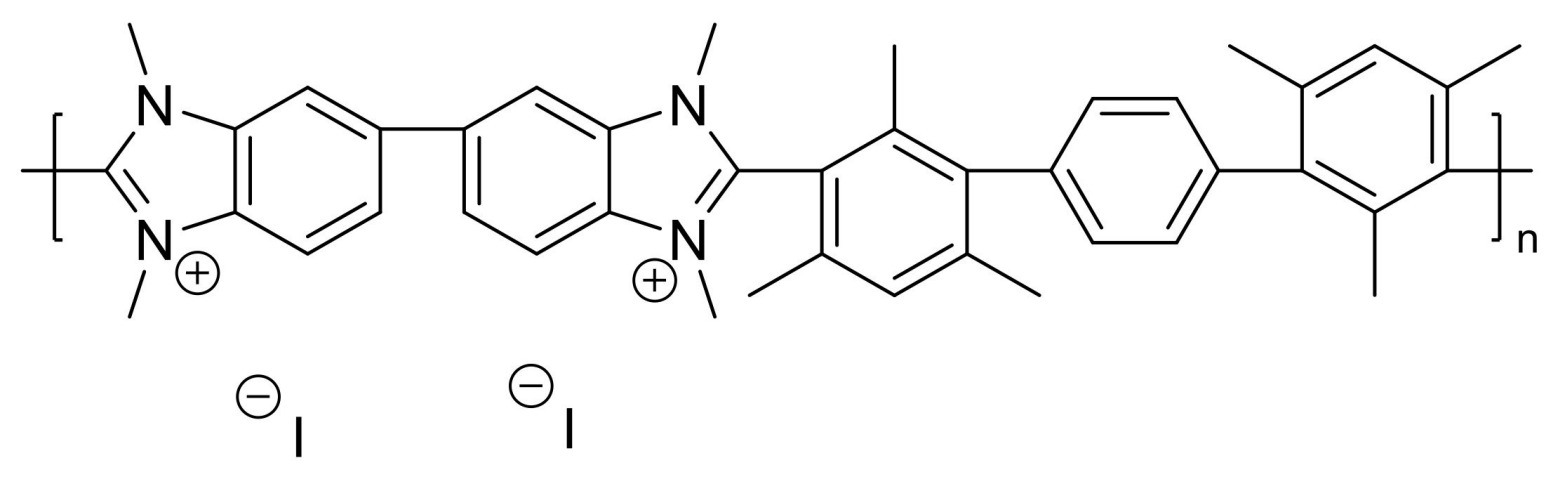
HMT-PMBI in the iodide form.

2,2”,4,4”,6,6”-hexamethyl-p-terphenylene-poly [2,2’-(m-mesitylene)-5,5’-bis(N,N’-methylbenzimidazolium)] (HMT-PMBI) was synthesized according to the procedure developed by Holdcroft et al. [36,57]. The as-prepared ionomer has a high degree of selectivity over the degree of methylation (dm %). The HMT-PMBI samples in this work are all of 92% dm unless stated otherwise. The polymer was received in a membrane form, with iodide (I^−^) as the counterion (see Scheme 1). In order to avoid potential interferences with the iodine form during the voltammetric studies, the counterion of HMT-PMBI was exchanged with chloride (Cl^−^), by soaking the membrane in 1 M NaCl for 24 h, then transferring it into DI water for 24 h.

### 2.3. Apparatus and Procedures

Cyclic voltammetry (CV), Differential pulse voltammetry (DPV), chronoamperometry (CA) and chronocoulometry (CC) were carried out using a potentiostat electrochemical analyzer (CH Instrument, Austin, TX, USA Model CHI760e). A three-electrode cell setup was used: a glassy carbon electrode (GCE) (CHI Instruments) with 3 mm diameter was employed as a working electrode, Ag/AgCl (3 M) was used as a reference electrode, and a platinum wire was used as a counter electrode. All experiments were carried out in a temperature-controlled room kept at roughly 20 °C. GCEs were cleaned using methanol and medical paper to ensure the surface was clean before polishing in a figure of eight motion, to gain a mirror-like appearance using 0.3 μm and 0.05 μm alumina slurry on microcloth pads (Buehler) for 5 min per grade of polishing pad, 15 min in total. The cleaning procedure was carried out immediately prior to each use or polymer deposition. Film thicknesses were measured using a Taylor Hobson, (Leicester, UK), Talysurf stylus profilometer, while pH measurements were done using a Hanna Instruments (Bedfordshire, UK) 2002 Edge pH meter.

### 2.4. HMT-PMBI Chemically Modified Electrodes

Different wt % solutions were prepared by dissolving a HMT-PMBI membrane in methanol. Five milligrams of HMT-PMBI were dissolved in 0.625 mL of methanol to obtain a 1% HMT-PMBI solution; 0.5% and 0.75% solutions were prepared by diluting the 1% solution. Ionene films were prepared by drop-casting on polished GCE surfaces a 5 µL aliquot of HMT-PMBI using an Eppendorf, (Hamburg, Germany) micropipette and then leaving the solvent to evaporate; the typical drying time was 10 min. HMT-PMBI films were loaded in K_4_Fe(CN)_6_ or K_2_IrCl_6_ redox mediators containing 0.1 M NaCl as a supporting electrolyte. All HMT-PMBI-coated electrodes were loaded in the redox mediator solution to ensure the complete saturation of the HMT-PMBI film. The ionene-coated electrodes loaded with the redox mediator were rinsed briefly with distilled water and transferred to an electrochemical cell containing only a supporting electrolyte solution, where CVs were carried out at a different range of scan rates. The surface coverage values were experimentally calculated from the charge, *Q*, associated with the complete oxidation of the film-bound redox species. *Q* was extracted by graphical integration of the background corrected cyclic voltammograms at a scan rate of 1 mV·s^−1^ in 0.1 M NaCl supporting electrolyte. The concentration of redox mediator incorporated into HMT-PMBI films was calculated from thickness measurements and the calculated surface coverage.

### 2.5. Detection of Uric (UA) and Ascorbic (AA) Acid

A 10 mM uric acid pH 7 stock solution was prepared by dissolving 0.038 g uric acid sodium salt in 20 mL deionized (DI) water containing 0.1 M NaCl as the supporting electrolyte. To facilitate the dissolution, the uric acid salt in the NaCl solution was constantly stirred and heated until it reached 40 °C. A 10 mM ascorbic acid stock solution at pH 4 was prepared by dissolving 0.0352 g of L-ascorbic acid in 20 mL background electrolyte 0.1 M NaCl. All other solutions were obtained by dilution of the 10 mM uric acid and ascorbic acid solutions. For pH experiments, the solution was adjusted as appropriate by the addition of concentrated HCl or NaOH solutions under stirring. For the analysis of uric acid in Surine^®^, the synthetic urine was considered to have all the constituents of a real urine sample, so no adjustment was made to the Surine^®^ solution, which has a pH of 6.8. Analysis of ascorbic acid in real samples was performed using commercial vitamin C tablets. For instance, 0.826 g of a Vitamin C tablet was dissolved in 100 mL DI water containing 0.1 M NaCl to give a concentration of ascorbic acid of 5.33 mM. Lower concentrations of Vitamin C were obtained after dilution of the initial batch as appropriate. The investigation into potential interference was carried out by the addition of various analytes to the 0.1 M solution of NaCl supporting electrolyte at 40 s time intervals, while running a chronoamperometry experiment with applied potential of 0.6 V under constant stirring. HMT-PMBI-coated electrodes were conditioned in Surine^®^ samples for 10 min with stirring. CV scans were carried out at a scan rate of 50 mV·s^−1^, E_initial_ −0.1 V; E_final_ 1 V, and DPV scans were carried out with increment 0.005 V, amplitude 0.05 V, pulse width 0.05 s, sampling width 0.0167 s, pulse period 0.5, E_initial_ −0.2 V; E_final_ 0.8 V.

## 3. Results and Discussion

### 3.1. General Electrochemical Properties

**Figure 1:**
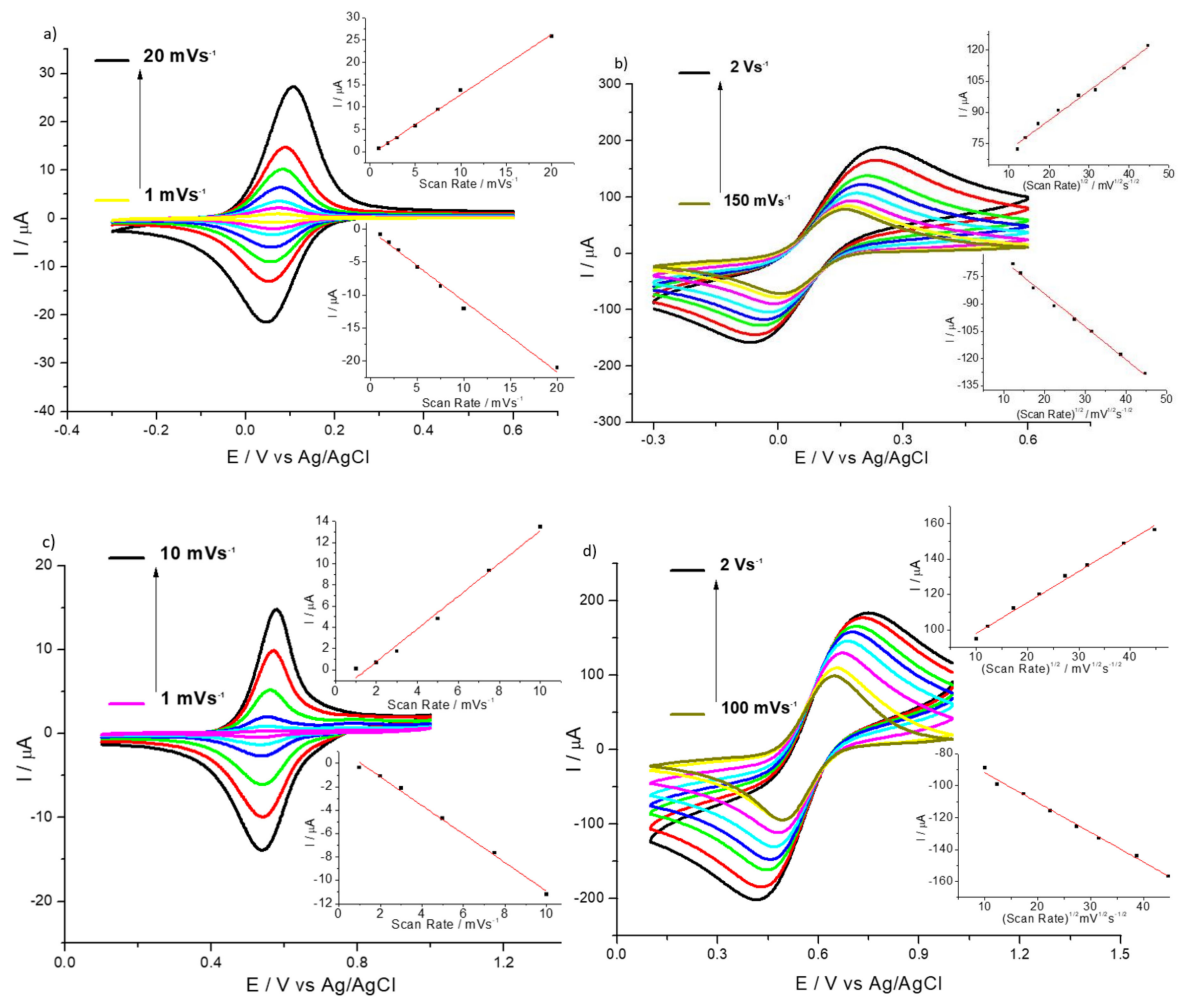
CVs of 1% HMT-PMBI-coated film loaded in 5 mM K_4_Fe(CN)_6_ (**a**,**b**) and 3 mM K_2_IrCl_6_ (**c**,**d**) after transferring to 0.1 M NaCl supporting electrolyte. Scan rate: from 1 mV s^−1^ to 20 mV s^−1^ (**a**), 1 mV s^−1^ to 10 mV s^−1^ (**c**), from 150 mV s^−1^ to 2 V s^−1^ (**b**) and 100 mV s^−1^ to 2 V s^−1^ (**d**). The inset graphs show the plot of the anodic and cathodic peak currents vs. the scan rate (**a**,**c**) and square root of the scan rate (**b**,**d**).

HMT-PMBI-coated electrodes were loaded into negatively charged redox mediators such as K_4_Fe(CN)_6_ and K_2_IrCl_6_ in order to establish whether the positively charged ionene films can incorporate species of the opposite charge. For instance, 1% HMT-PMBI-modified electrodes were loaded into a 5 mM or 3 mM solution of K_4_Fe(CN)_6_ and K_2_IrCl_6_, respectively. Figure S1 shows the corresponding voltammograms recorded during loading conditions with the appearance of the typical redox peaks of Fe^2+/3+^ (Figure S1a) and Ir^4+/3+^ redox couples (Figure S1b). The incorporation of the redox mediator occurs rapidly for both redox mediators, with the peak currents reaching constant values after ca. 15 min, an indication of the saturation of the coated electrode. The CVs recorded under loading conditions depict the quasi-reversible redox behavior for the Fe(CN)_6_^4−/3−^ and IrCl_6_^2−/3−^ redox couples, with peak currents scaling linearly with the square root of the scan rate and peak-to-peak separation higher than 59 mV, as expected for a diffusion-controlled process. When the loaded electrodes were transferred to only supporting electrolyte (see Figure 1a–d), the peak currents decreased to 64 μA from a value of 92 μA for Fe(II) and to 93 μA from the value of 156 μA for Ir(IV) initially recorded in the loading solutions. A detailed analysis of the CVs of HMT-PMBI-coated electrodes reveals that, at low scan rates (v < 10 mV s^−1^), the peak current scales linearly with the scan rate, with ΔE_p_ values between 11 mV and 51 mV for Fe(II) and 19 mV and 38 mV for Ir(IV) and well below the value of 59 mV expected for a Nernstian process. This behavior is typical of a thin-layer or surface-controlled process. A surface-controlled process is operative when the polymer layer is thinner than the concentration gradient of the redox species, and therefore, as expected, the transition from surface- to diffusion-control is also thickness-dependent [58]. Instead, for scan rates higher than v > 150 mV s^−1^, for Fe(II) and v > 100 mV s^−1^ for Ir(IV), the ΔE_p_ values increase monotonically and the peak currents scale linearly with the square root of scan rate indicating a diffusion-controlled process [58]. It is important to note that in the time scale of the experiment (20 min) the loss of the redox mediators incorporated in the film was around 25%.

By integration of the oxidation or reduction peak currents, recorded at the low scan at in which exhaustive electrolysis takes place, it is possible to calculate the charge and then estimate the concentration of the redox species incorporated within the film with the knowledge of the film thickness. The detailed procedure is shown in Figure S2. The concentration of the redox mediator within the film, substituted into the Randles‒Sevcik equation, allowed for the estimation of the apparent diffusion coefficient, D_app_. The calculated D_app_ values for Fe^2+/3+^ and Ir^4+/3+^ redox couple using the Randles‒Sevcik equation were found to be (6.4 ± 0.10) × 10^−10^ cm^2^·s^−1^ and (2.72 ± 0.85) × 10^−9^ cm^2^·s^−1^, respectively, for 1% HMT-PMBI-coated electrodes. Instead, the values of D_app_ using the Anson’s method were estimated as (2.40 ± 0.6) × 10^−10^ cm^2^·s^−1^ and (3.11 ± 1.24) × 10^−9^ cm^2^·s^−1^. These values agree with those calculated using the Randles‒Sevcik equation and are in the range of those D_app_ reported in the literature for other ionomers [22,25,26,27,28,29,32]. Note that, in using these methods to evaluate the apparent diffusion coefficients, we made the assumptions that HMT-PMBI films do not swell when immersed in solution and that the ionene coating is uniformly distributed across the GCE electrode surface. Swelling of ionomer films during the voltammetric scan is a well-known effect, as shown by Bard and Anson [29,32], and likely to occur in ionene films too. This effect is ascribed to the fact that during the forward and backward scans there is ingress and egress of ions of opposite charge traveling throughout the polymer film in order to maintain the electroneutrality. Hence, the values of D_app_ reported here ought to be taken as a general estimation. It is interesting to note that the estimated values of D_app_ for the IrCl_6_^2−/3−^ are ca. one order of magnitude higher than those of the Fe(CN)_6_^4−/3−^ redox couple. This may be due to the fact that the tertiary amine in HMT-PMBI is more effective at preconcentrating a redox mediator bearing a 2^−^/3^−^ charge as for the potassium hexachloroiridate rather than a 4^−^/3^−^ of the potassium ferrocyanide. This underlines the fact that Fe(CN)_6_^4−/3−^ specie is an inner-sphere and surface-sensitive redox mediator, whereas IrCl_6_^2−/3−^ is an outer-sphere and surface-inert one. Nonetheless, the values of D_app_ are within the expected range for ionomer-coated films, such as Nafion^®^ [32] and TPQPCl [22]. Table 1 summarizes the values of the surface coverage, concentration of redox probe, and apparent diffusion coefficients extracted using these methods for 0.5%, 0.75% and 1% HMT-PMBI-coated electrodes. It is worth noting that HMT-PMBI-coated films retain the redox species after being transferred to a supporting electrolyte solution, as evidenced by the concentration of the redox mediator inside the film, on the order of magnitude of 10^−2^‒10^−1^ mol·dm^−3^.

**Figure 2:**
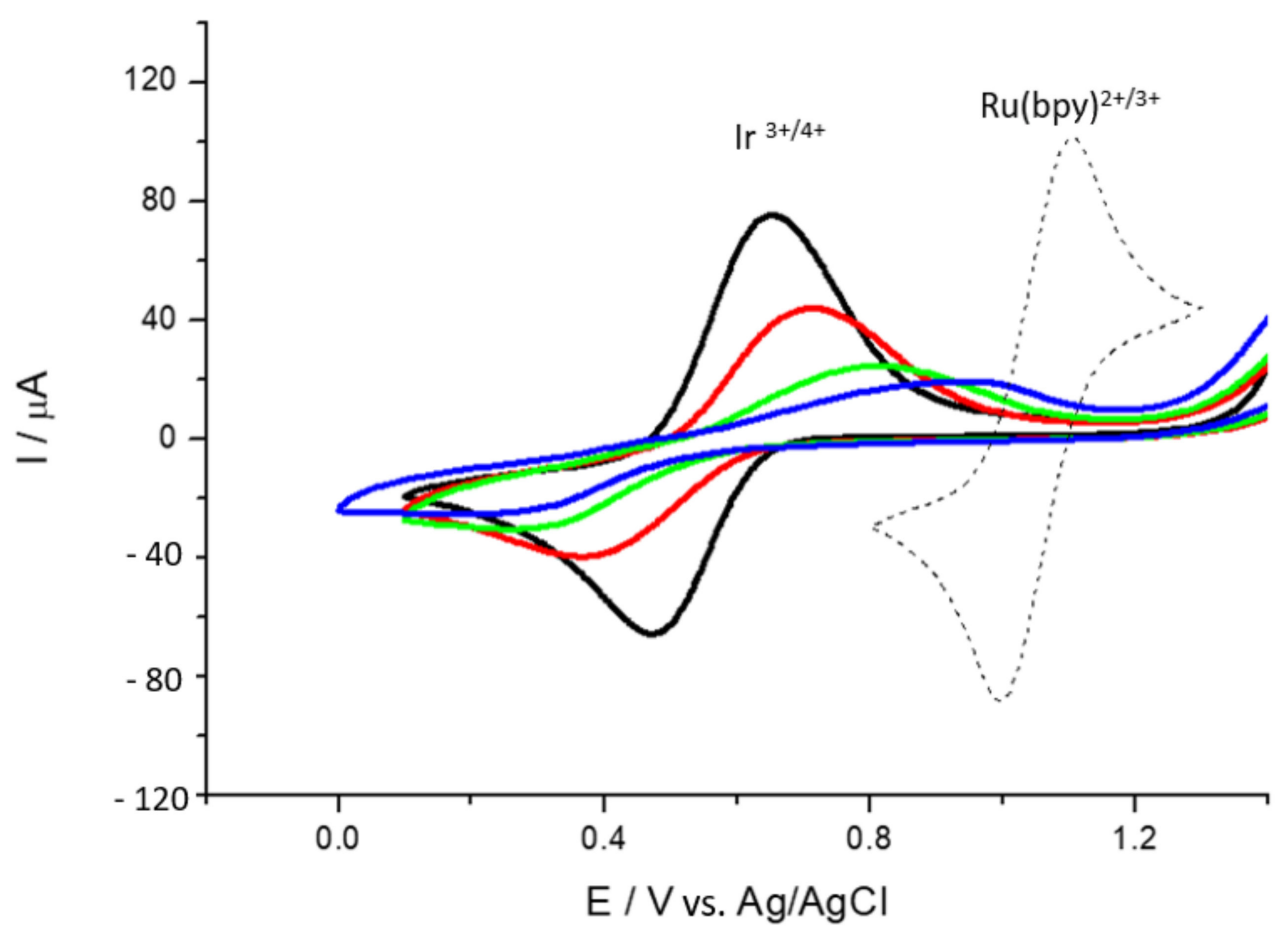
CVs of 1% HMT-PMBI-coated film loaded in 3 mM K_4_IrCl_6_ after transferring to 0.1 M NaCl supporting electrolyte, and with the addition of different concentrations of Ru(bpy)_3_^2+^: 0.1 mM (black); 5 mM (red), 15 mM (green) and 30 mM (blue). Dashed CV: bare GCE recorded in 10 mM Ru(bpy)_3_^2+^; scan rate of 100 mV·s^−1^.

Noticeably, HMT-PMBI-coated films retain a significant proportion (ca. 53%) of Fe(II) and (ca. 15%) of Ir(IV) of the redox mediator, and despite the peak current decreasing over time, a voltammetric peak is still clearly distinguished even after 1 h of continuous cycling in only supporting electrolyte (see Figure S3). It is clearly visible that iridium is retained much less than iron, in agreement with the fact that D_app_ for Ir^4+/3+^ is higher than the D_app_ for Fe^2+/3+^. An important feature of ion-exchange membranes is the permselectivity, e.g., their ability to incorporate ions of opposite charge while at the same time repelling species of the same charge. Figure 2 shows the CVs recorded for 1% HMT-PMBI-coated electrodes fully loaded in 3 mM of K_2_IrCl_6_ after being transferred to a supporting electrolyte containing different concentrations (from 0.1 mM to 30 mM) of a positively charge redox probe such as Ru(bpy)_3_^2+^. The HMT-PMBI-coated electrode does not show any voltammetric peak related to the redox behavior of the Ru(bpy)_3_^2+/3+^ couple until the concentration reaches 15 mM. Noticeably, the peak current related to the Ir^4+/3+^ decreases as the concentration of Ru(bpy)_3_^2+^ increases, whereas the CVs show a more irreversible behavior, with the ΔE_p_ values increasing from 180 mV before the addition of Ru(bpy)_3_^2+^ to 702 mV as the concentration of Ru(bpy)_3_^2+^ reaches 30 mM. The CV recorded at 30 mM concentration of Ru(bpy)_3_^2+^ shows only a feeble peak related to the oxidation of Ru^2+/3+^ at ca. 1 V when compared to the CV of the bare GCE in the Ru(bpy)_3_^2+^ solution, which is barely distinguishable from the peak related to the oxidation of Ir^3+^ to Ir^4+^. This effect is not surprising considering that the higher concentrations of Ru(bpy)_3_^2+^ displace IrCl_6_^2−^ ions incorporated within the film. It is interesting to note that at higher (30 mM) concentrations of Ru(bpy)_3_^2+^ only the oxidation peak is visible, whereas there is no trace at all of the reduction peak as an indication that Ru(bpy)_3_^3+^ ions are ejected even more efficiently by the HMT-PMBI coating.

### 3.2. Detection of Uric and Ascorbic Acids in HMT-PMBI-Coated Electrodes

**Scheme 2:**
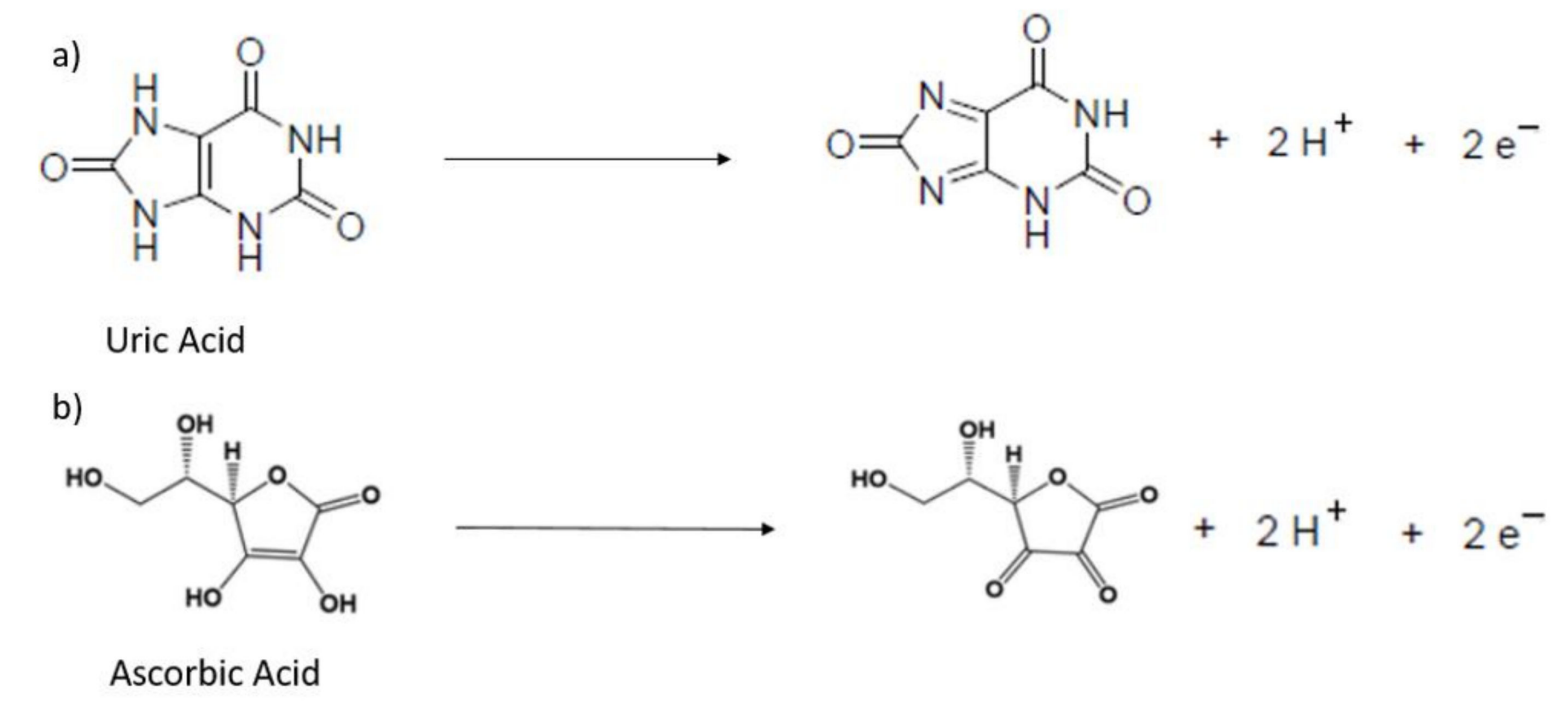
Mechanism of oxidation for uric acid (**a**) and ascorbic acid (**b**).

**Figure 3:**
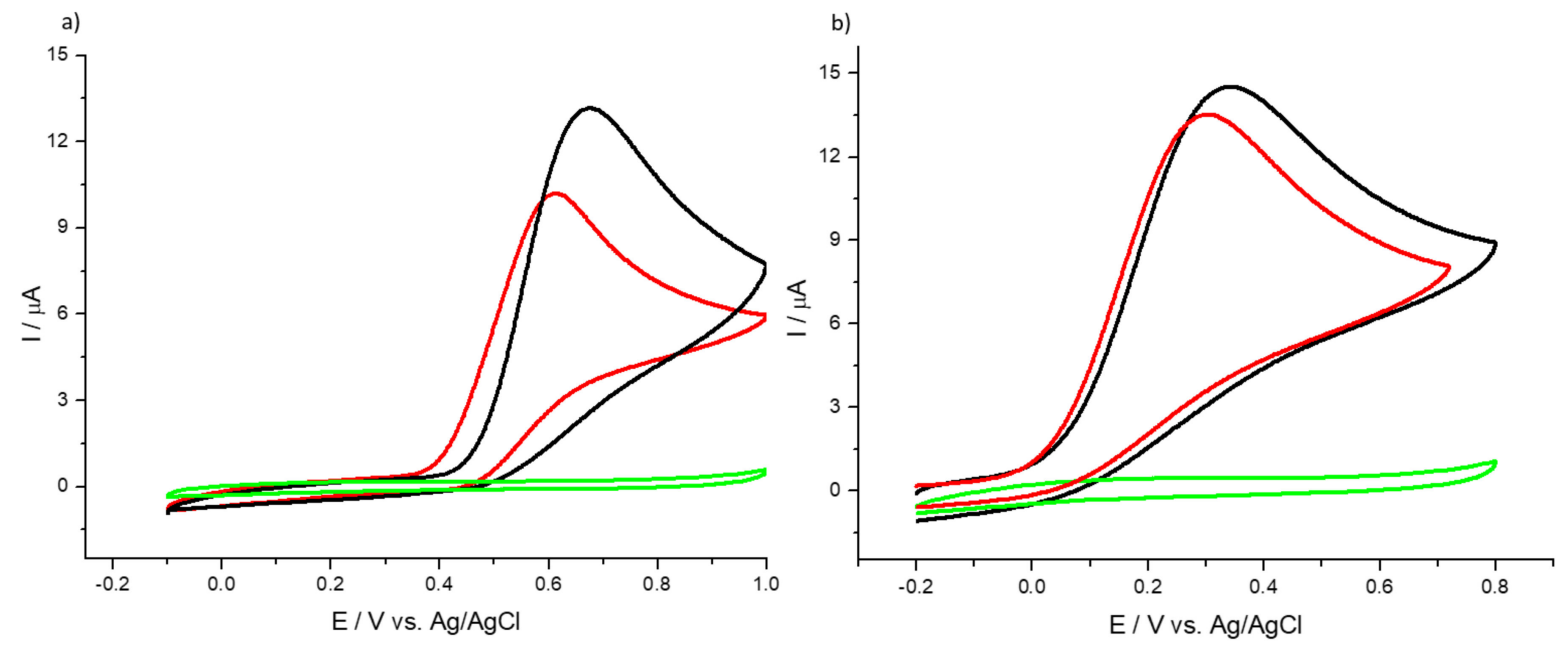
CVs of bare GCE (red) and 1% HMT-PMBI-coated electrode (black) recorded in 1 mM UA at pH 7 (**a**) and 1 mM AA at pH 4 (**b**); supporting electrolyte: 0.1 M NaCl; scan rate of 50 mV·s^−1^. The curve in green represents the blank CV of GCE recorded in supporting electrolyte only.

In order to ascertain the suitability of the as-prepared ionene films for electrocatalytic sensing studies, we investigated the electrochemical behavior of HMT-PMBI towards the detection of uric (UA) and ascorbic acid (AA). These studies were performed using cyclic voltammetry, chronoamperometry and differential pulse voltammetry. Figure 3 depicts the CVs recorded at bare GCE and 1% HMT-PMBI-coated electrodes in the presence of 1 mM uric acid. The oxidation of UA occurs at 615 mV for the bare GCE, whereas it shifts to a more positive potential (to 676 mV vs. Ag/AgCl) in the case of the 1% HMT-PMBI-coated electrode. This effect is not surprising and typical of ionomers deposited on electrode surfaces, in which the overall diffusion is given by two contributions, e.g., (1) the diffusion of the electroactive specie from the bulk solution to the ionomer coating, and (2) the much slower diffusion of the electroactive specie from the ionomer coating to the GCE surface [59]. This means that more energy is required to oxidize the electroactive species on the electrode surface; this leads to a shift towards a more positive potential. When the loaded electrode is transferred to the supporting electrolyte, the peak potential shifts to a more negative potential as there is only the contribution from the diffusion within the ionomer coating. The CVs show the typical peak current related to the two-electron, two-proton irreversible oxidation of UA via the formation of an unstable bis-imine specie, which further hydrolyses to 4,5-diol uric acid, as shown in Scheme 2a [60,61,62].

In the case of ascorbic acid (Figure 3b), the CVs show an oxidation peak at 307 mV, typical of the irreversible oxidation of ascorbic acid to dehydroascorbic acid (see Scheme 2b).

**Figure 4:**
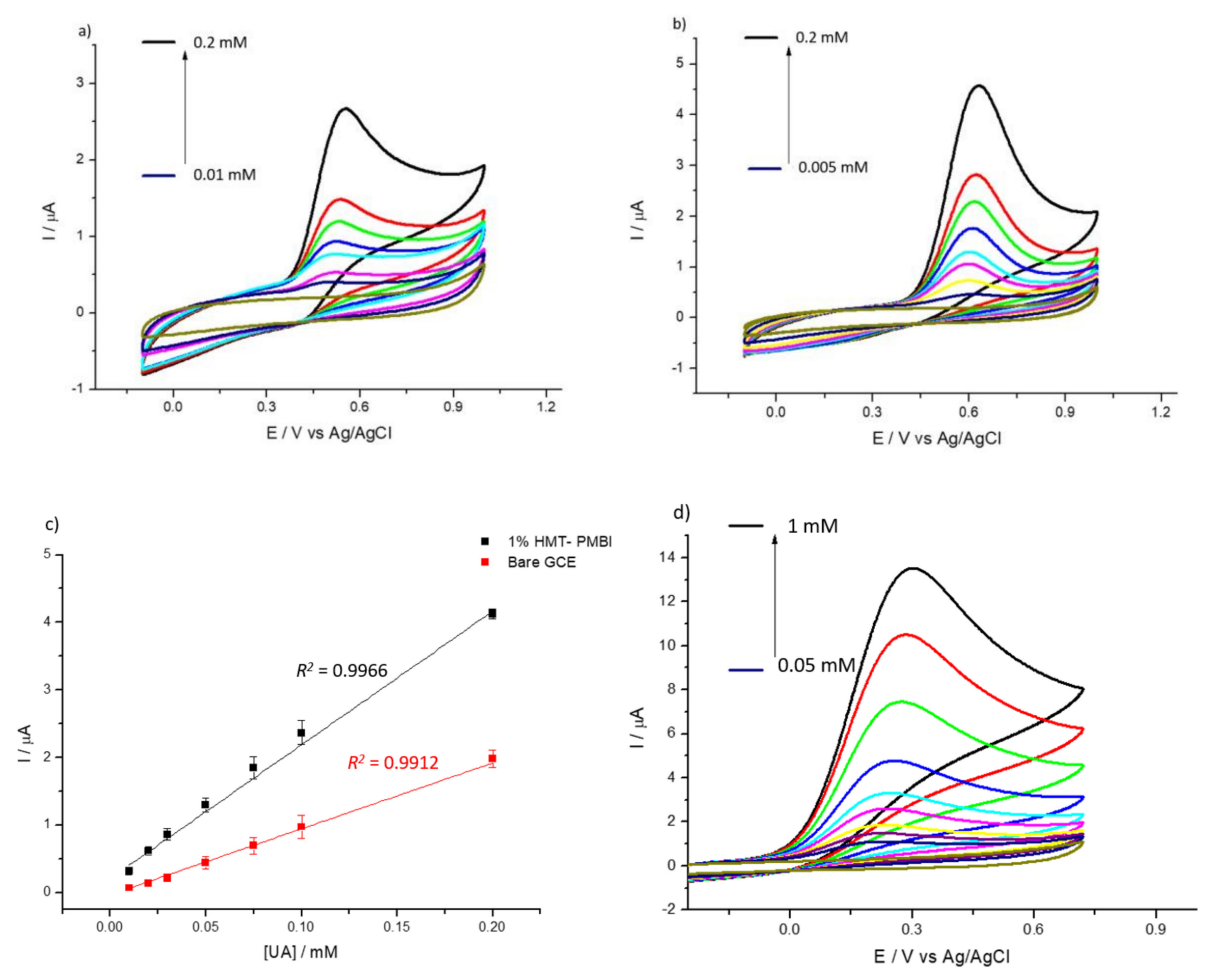

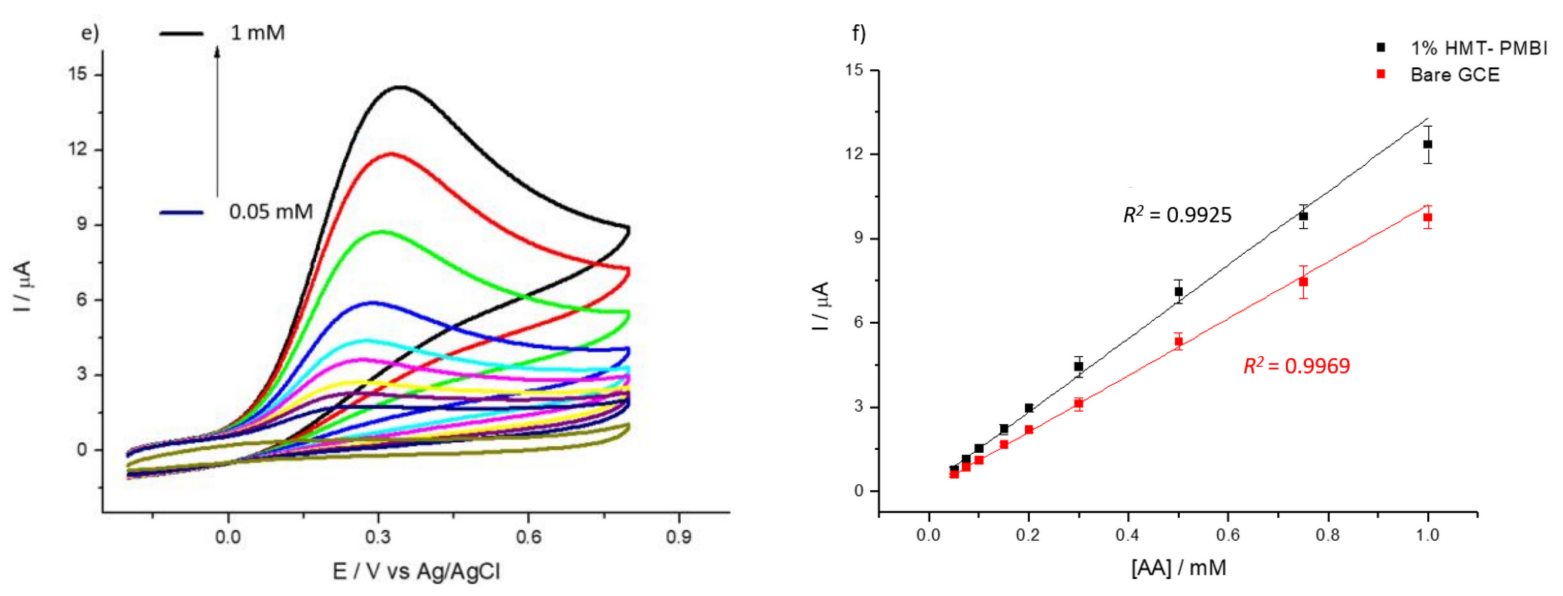
CVs of bare GCE (**a**,**d**) and of 1% HMT-PMBI (**b**,**e**)-coated electrode recorded in 0.1 M NaCl supporting electrolyte in the presence of various concentrations of UA from 5 μM to 0.2 mM (**a**,**b**) and AA from 5 μM to 1 mM (**d**,**e**). Scan rate of 50 mV s^−1^. Plot of peak currents vs. concentration of UA (**c**) and AA (**f**); bare GCE (red line), HMT-PMBI-coated electrode (black line).

Note the higher peak current for the coated electrode—as an indication of the ionene capability to preconcentrate uric and ascorbic acid. Similar to the incorporation of the redox mediators, the HMT-PMBI-coated films can retain UA and AA when transferred to supporting electrolyte only (see Figure S4). The CVs recorded in 1 mM uric acid at a different loading time from the 1% HMT-PMBI-coated electrodes show that the voltammetric signal plateaus after 15 min loading time. This loading time was then selected for all further experiments (see Figure S5). Also, we investigated the effect of the pH on the voltammetric behavior of both UA and AA. Figure S6 shows the voltammetric response related to 1 mM uric acid (S6a) and 1 mM ascorbic acid (Figure S6b) obtained at different values of pH. In the case of UA, at pH 4, the oxidation peak occurs at ca. 0.72 V, whereas with the increase of the pH the oxidation peak shifts to a less positive potential (ca. 0.61 mV) at neutral and basic pH values, in agreement with previous reports [63,64]. Note also the concomitant increase of the peak current from 12 μA at pH 4 to ca. 15 μA at higher pH values (7–10). At neutral and basic pH, the peak current is almost constant. In the case of AA, the highest peak current is obtained at pH 4 and with the increase in the pH, the peak currents decrease considerably. This dramatic decrease in the peak currents at higher pH values (pH > 4) is due to the fact that deprotonation (see Scheme 2b) is involved in the electro-oxidation of AA, which is facilitated at lower (acidic) pH values, as reported by other authors [65,66,67]. As a result of that, we performed all further experiments involving UA and AA at pH 7 and pH 4, respectively. Figure 4 reports the CVs obtained for the bare GCE (Figure 4a) and the 1% HMT-PMBI-coated electrodes (Figure 4b) recorded at different concentrations of uric acid from 0.01 mM to 0.2 mM. In both cases, the peak current increases linearly with the concentration; however, for the HMT-PMBI-coated electrode there is an enhancement of the peak current compared to the bare GCE that becomes even more pronounced at high concentrations of uric acid. The corresponding plot of the anodic peak current vs. uric acid concentration (Figure 4c) exhibits a linear response in the range from 0.01 mM to 0.2 mM with a regression equation expressed as I_p_ (μA) = 0.02 (μA μM^−1^) [UA] (μM) + 0.2 (μA) (*R*^2^ = 0.9966). Hence, the sensitivity of HMT-PMBI-coated electrodes is calculated as 0.28 µA·µM^−1^·cm^−2^, whereas the limit of detection (LoD) calculated from the slope, S, of the linear regression and the standard deviation S_B_ of three repeats of 1 mM uric acid determination using the relation LoD = 3S_B_/S, is 18 μM. Figure 4d,e depicts the data obtained at different concentrations of AA from 0.05 mM to 1 mM for the GCE and HMT-PMBI, respectively. Also, in this case the corresponding plot of the anodic peak current scales linearly with the ascorbic acid concentration with a regression equation as I_p_ (μA) = 0.013 (μA μM^−1^) [AA] (μM) + 0.23 (μA) (*R*^2^ = 0.9925) (Figure 4f). The calculated sensitivity and LoD of the HMT-PMBI-coated electrode are 81 µA·µM^−1^·cm^−2^ and 0.18 μM, respectively.

**Figure 5:**
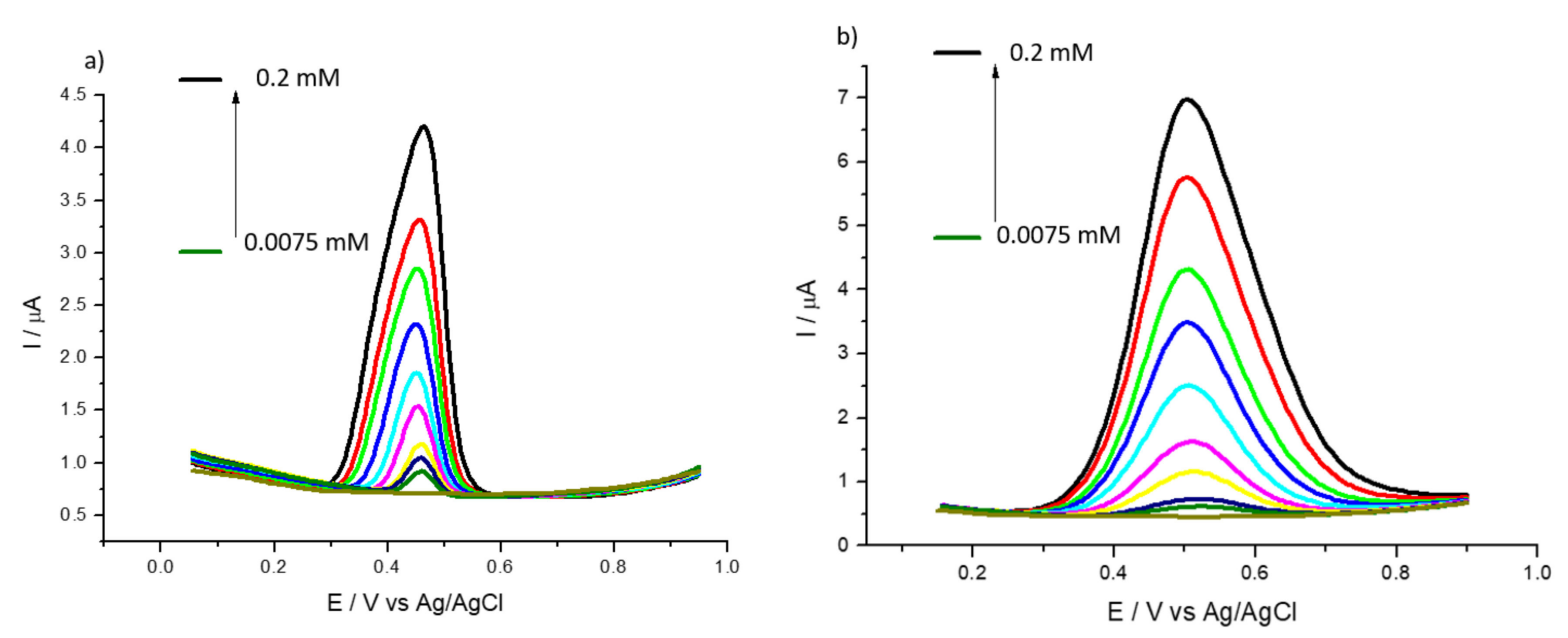

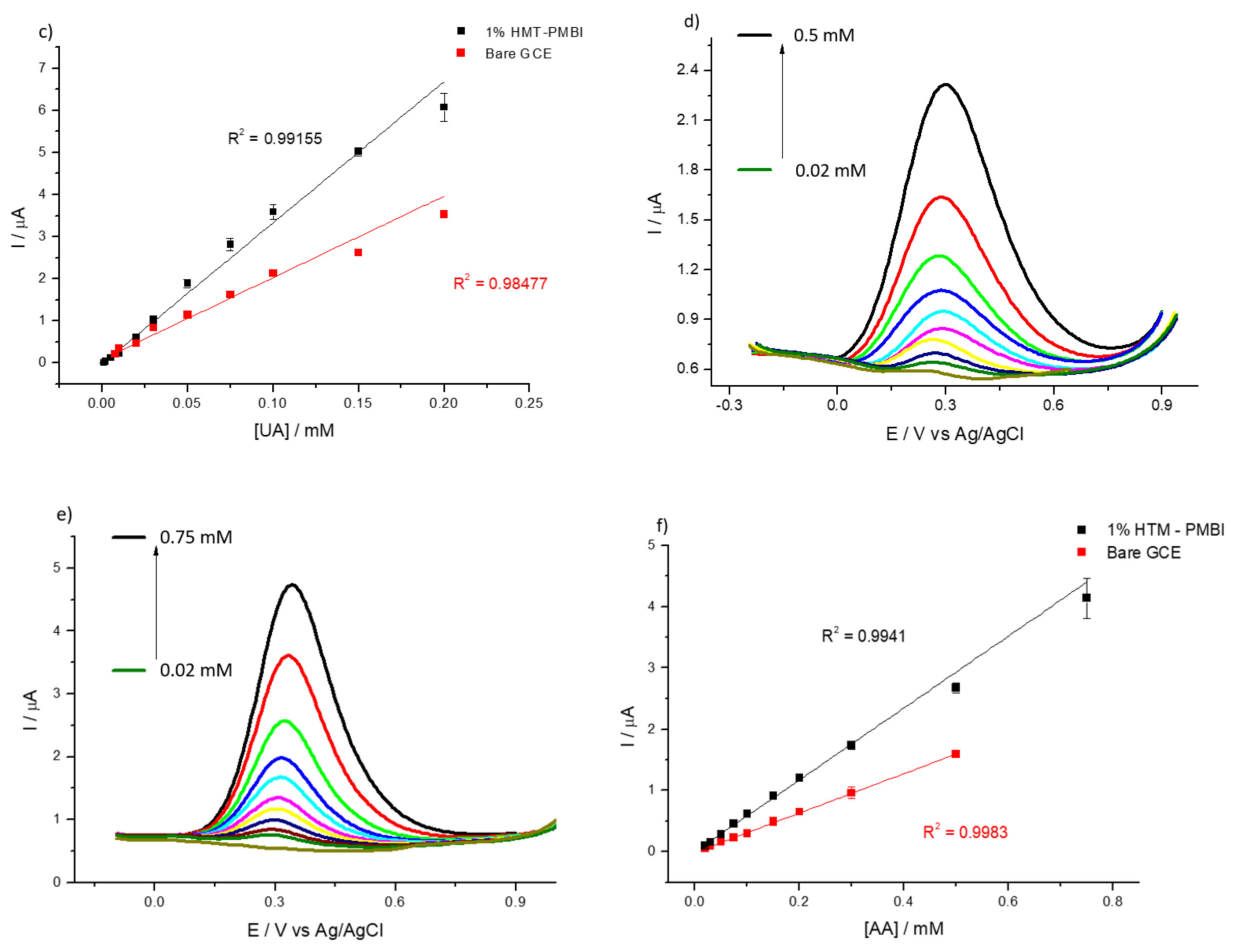
DPVs of bare GCE (**a**,**d**) and of 1% HMT-PMBI (**b**,**e**)-coated electrode recorded in 0.1 M NaCl supporting electrolyte in the presence of various concentrations of UA from 7.5 μM to 0.2 mM (**a**,**b**) and AA from 20 μM to 0.75 mM (**d**,**e**). Scan rate of 10 mV s^−1^. Plot of peak currents vs. concentration of UA (**c**) and AA (**f**); bare GCE (red line), HMT-PMBI-coated electrode (black line).

The electrochemical behavior of bare GCE and 1% HMT-PMBI-coated electrodes was investigated using differential pulse voltammetry as well. Figure 5 shows the DPVs recorded in the presence of UA. Figure 5a,b show the typical oxidation peak of uric acid at 0.44 V and 0.50 V for the bare GCE and HMT-PMBI-coated electrode, respectively. In the case of detection of ascorbic acid (Figure 5d,e), the oxidation peaks appear at 0.28 V and 0.30 V for the bare GCE and HMT-PMBI, respectively. Notably, the peak currents recorded for the HMT-PMBI-coated electrodes are higher than those for the bare GCE, further confirming the suitability of the ionone to preconcentrate negatively charged species.

**Figure 6:**
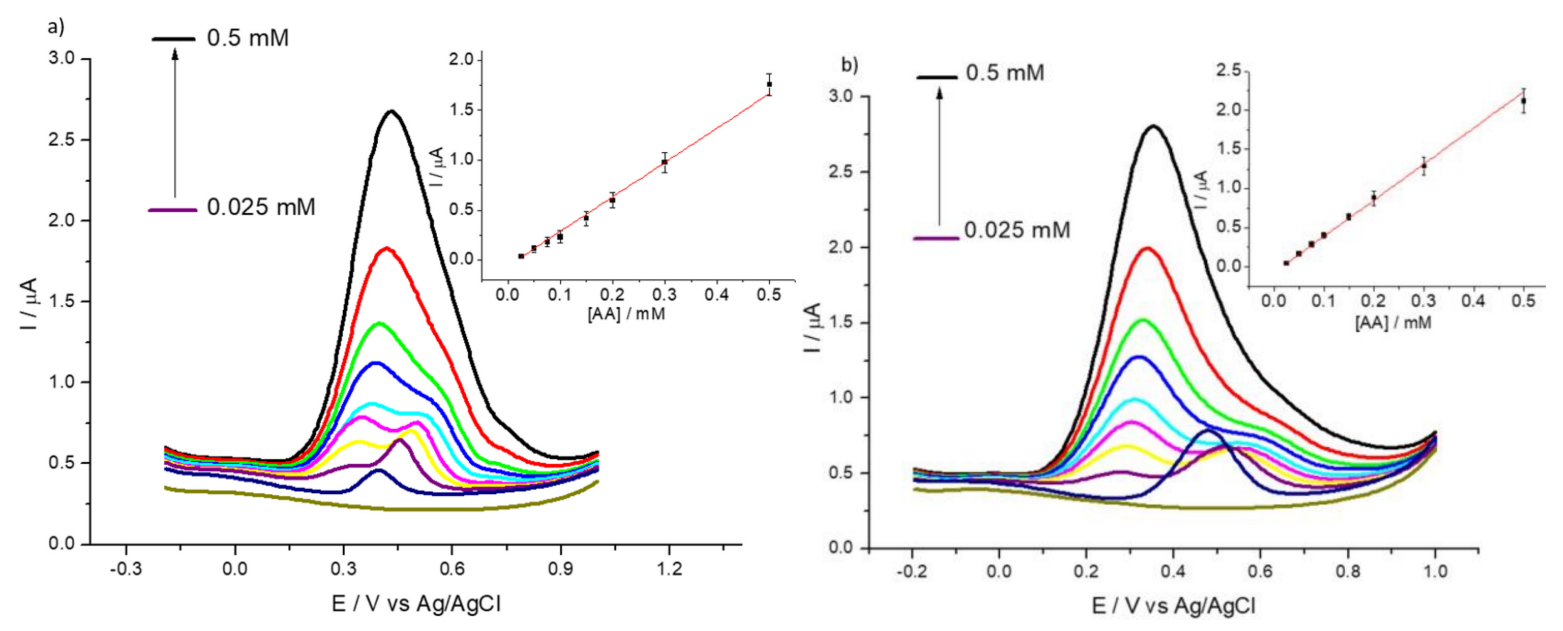
DPVs of bare GCE (**a**) and of 1% HMT-PMBI (**b**)-coated electrode recorded in 0.1 M NaCl supporting electrolyte in the presence of constant 0.01 mM UA and various concentrations of AA, from 25 µM to 0.5 mM. Scan rate of 10 mV·s^−1^. Inset: plot of peak currents vs. concentration of AA.

It is well known that the detection of uric and ascorbic acids is often made difficult by the fact that both species oxidize at very similar potentials, so the two species tend to interfere with each other. For this purpose, we recorded DPVs in order to ascertain the voltammetric behavior of HMT-PMBI in the presence of both UA and AA. Figure 6 shows the DPV curves for the bare GCE (Figure 6a) and 1% HMT-PMBI (Figure 6b) recorded at a constant concentration of uric acid (0.01 mM) and with increasing concentrations of ascorbic acid from 25 µM to 0.5 mM. The peak related to the oxidation of UA occurs at ca. 0.45 V vs. Ag/AgCl for the bare GCE with the appearance of the oxidation peak of AA at ca. 0.30 V.

**Figure 7:**
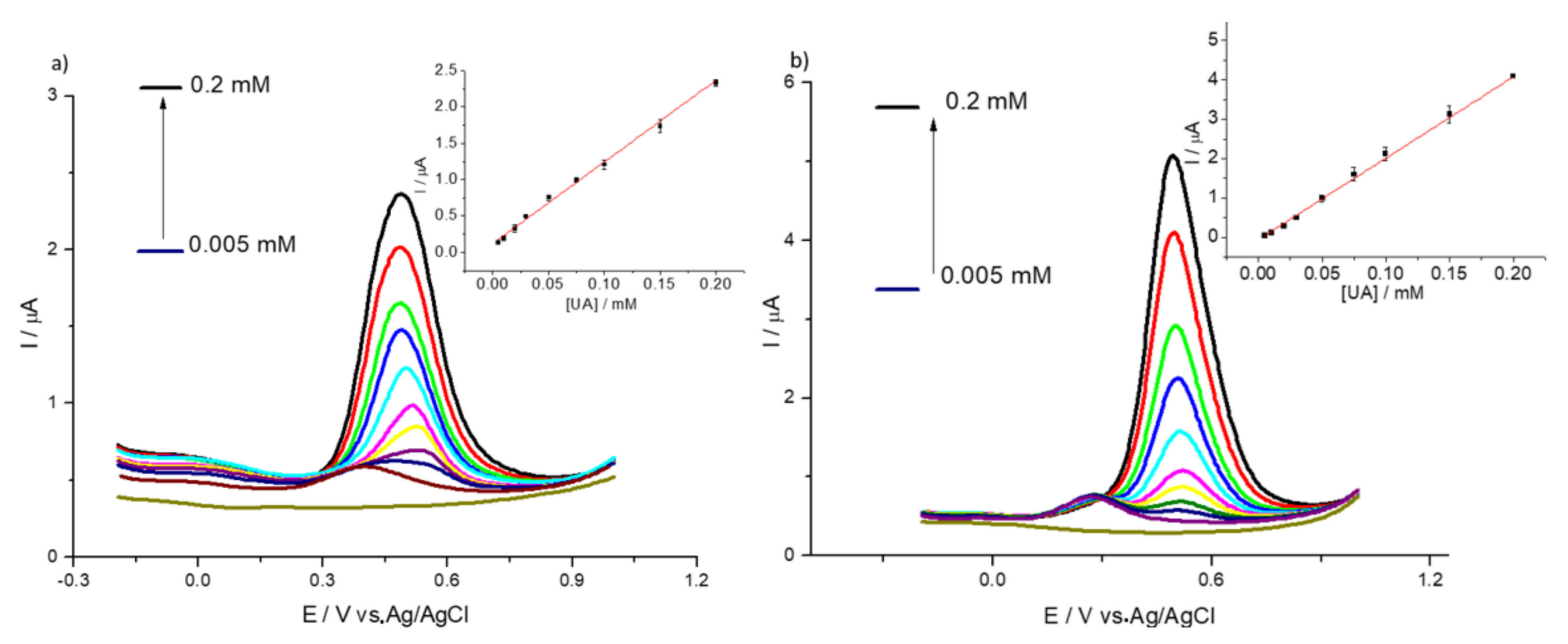
DPVs of bare GCE (**a**) and of 1% HMT-PMBI (**b**)-coated electrode recorded in 0.1 M NaCl supporting electrolyte in the presence of constant 0.05 mM AA and various concentrations of UA, from 5 µM to 0.2 mM. Scan rate of 10 mV s^−1^. Inset: plot of peak currents vs. concentration of UA.

In the case of 1% HMT-PMBI-coated electrodes, the oxidation peak of UA occurs at ca. 0.5 V and shifts to more positive potentials when compared to the bare GCE. However, the peak of AA is constant at 0.30 V and increases with the AA concentration. At higher concentrations of AA, the two peaks are virtually indistinguishable, hence the detection of UA in the presence of higher concentrations of AA is problematic. The same situation occurs when repeating the experiments, keeping constant the concentration of AA and adding different concentrations of UA, as reported in Figure 7. Again, while for the bare GCE it is very difficult to discriminate between the AA and UA contribution, for the 1% HMT-PMBI the two peaks are distinguishable and separated by a ΔE_p_ of ca. 210 mV.

**Figure 8:**
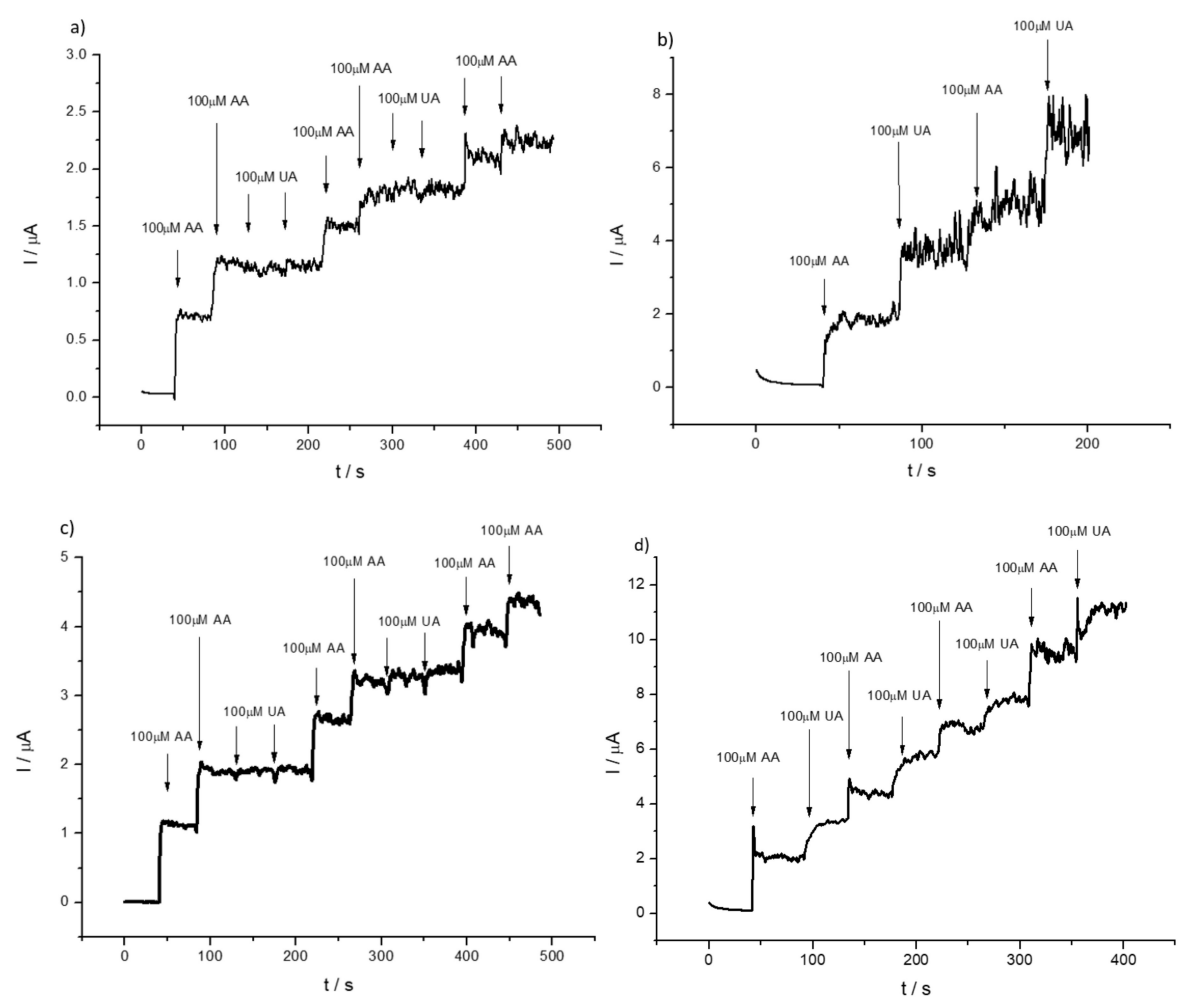

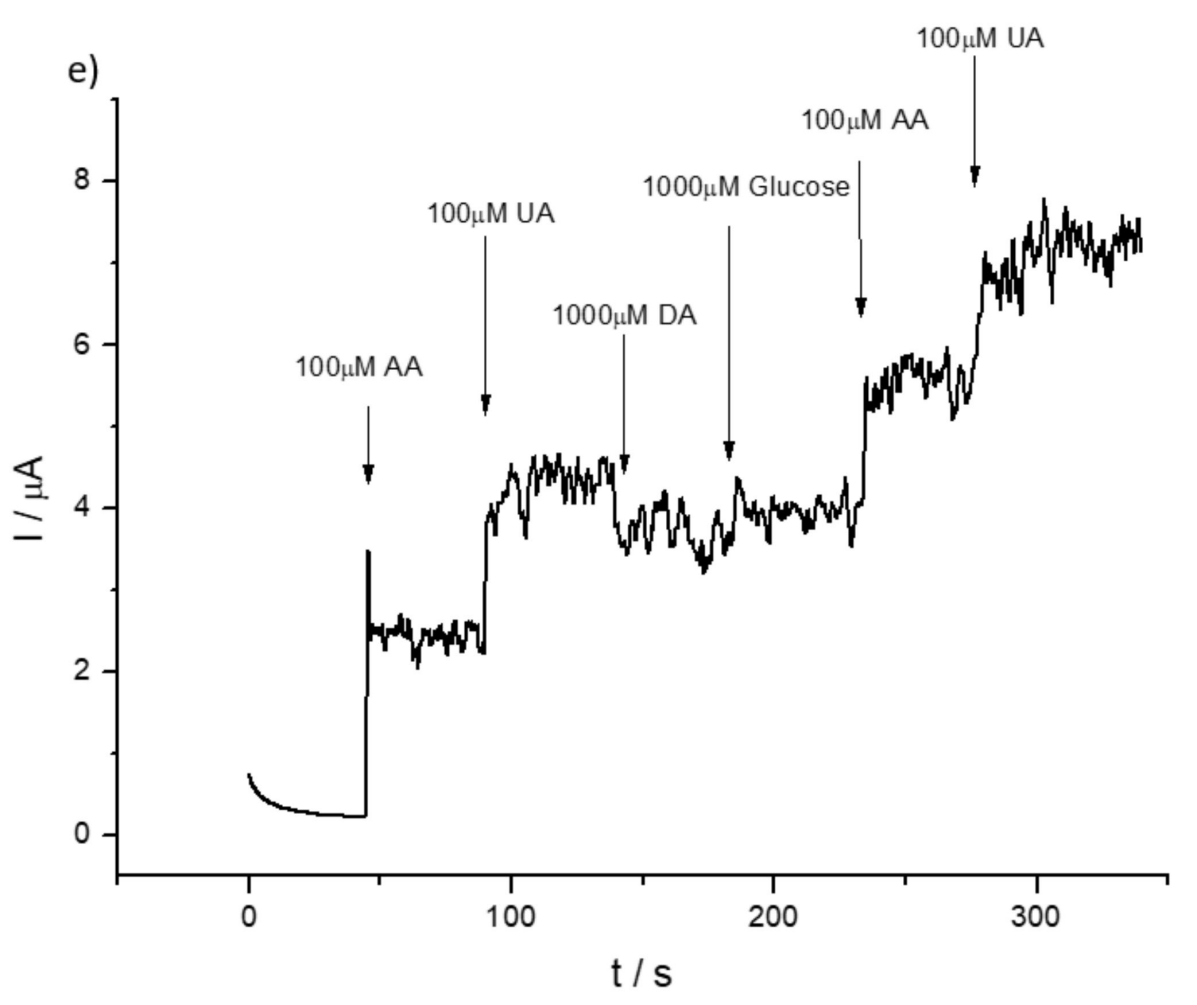
Chronoamperometric (i-t) response of bare GCE (**a**,**b**) and of 1% HMT-PMBI (**c**,**d**)-coated GCE applying 0.3 V and 0.6 V obtained with successive concentration of 100 µM UA and 100 µM AA from recorded in 0.1 M NaCl supporting electrolyte (pH 7) (**e**) 1% HMT-PMBI applying 1 V and adding concentrations of UA, AA, DA and glucose.

To underline the fact that the concentration of AA in physiological fluids is in the millimolar range and of the same order as that of UA, hence, as shown in Figure 7, interferences are very likely. A similar situation occurs in the case of detection of AA in the presence of dopamine, where the simultaneous detection of two species is problematic (see Figure S7a) for the bare GCE, whereas in the case of 1% HMT-PMBI-coated electrodes, the separation of the two peaks is more pronounced even though the identification of the two species becomes an issue at high concentrations of dopamine. Since dopamine is positively charged, this species is effectively repelled by the positively charged ionene, and very high concentrations are needed in order to be detected at the modified electrode (see Figure S7b). We point out that in physiological fluids the concentration of dopamine is much lower (on the order of 0.5–4.8 nM) [68] than that of ascorbic acid, hence the situation represented here is unrealistic. The analysis of the DPVs performed at 1% HMT-PMBI suggests that chronoamperometry could be useful for the quantification of both AA and UA, but with the requirement of performing the experiment at different applied potentials to avoid interferences. To begin with, we checked the chronoamperometric response of AA at bare GCE and 1% HMT-PMBI-coated electrodes. The CA response for AA obtained by applying 0.3 V is shown in Figure S8. The current recorded at the bare GCE and 1% HMT-PMBI-coated electrodes increases with the concentration of AA; however, the sensitivity is more pronounced for the ionene-coated electrode. Significantly, for the bare GCE the current plateaus at concentrations higher than 0.1 mM, whereas for 1% HMT-PMBI the current increases linearly up to a 0.2 mM concentration of AA. The plot of the oxidation current vs. the concentration of AA exhibits a linear response in the range from 5 µM to 0.2 mM with a regression equation I_p_ (µA) = 0.011 (μA μM^−1^) [AA] (µM) + 0.00014 (μA) (*R^2^* = 0.991) µA. Therefore, the sensitivity of HMT-PMBI-coated electrodes is calculated as 0.16 µA µM^−1^ cm^−2^ and the limit of detection, LoD, is 17 μM. The LoD is calculated from the slope, S, of the linear regression plot and the standard deviation S_B_ of three repeats of 0.1 mM AA addition, using the equation LoD = 3S_B_/S. In a similar way, the limit of quantification, LoQ is calculated as 52 µM from the plot of the linear regression plot S and the standard deviation S_B_ of 10 repeats of 0.1 mM AA addition using the equation LoQ = 10S_B_/S. In the case of detection of UA, CA were performed by applying a potential of 0.6 V. Figure S9 shows the corresponding CA curves at different UA concentrations for the bare GCE and 1% HMT-PMBI-coated electrodes. Note that the HMT-PMBI performs better at higher concentrations of uric acid, while there are no substantial differences with the bare GCE at low concentrations. The linearity range of values for UA is 5 µM to 0.3 mM, whereas the calculated LoD and LoQ are 6 µM and 20 µM, respectively. We point out the fact that in physiological fluids, the average concentration of UA is 200 µM, which is a relatively high [41]; hence, in this range we expect the HMT-PMBI to perform better than the bare GCE. Figure 8 shows the CA responses obtained for the bare GCE by applying a different potential of 0.3 V (a) and 0.6 V (b) after the successive addition of UA and AA, respectively. The corresponding CA for the 1% HMT-PMBI-coated electrode obtained in the same conditions are reported in Figure 8c,d. As expected, and based on the DPV data, at 0.3 V the current increases only with the addition of AA, whereas it is insensitive to UA, which oxidizes at a higher potential. Instead, at 0.6 V, both the bare GCE and 1% HMT-PMBI curves show an increase of the current with the increase in concentration of AA and UA. However, glucose (see Figure 8e) and dopamine do not show any interference even after application of 1 V.

**Figure 9:**
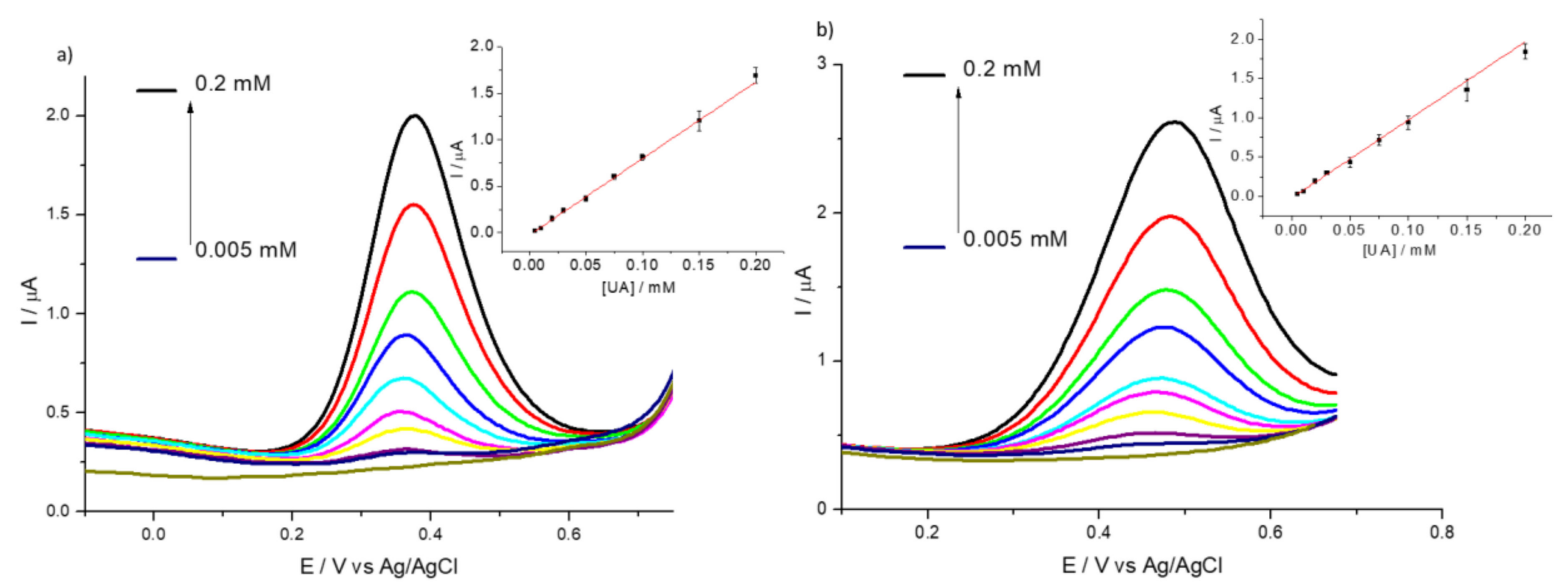
DPVs of bare GCE (**a**) and of 1% HMT-PMBI-coated electrode (**b**) recorded in Surine^®^ in the presence of various concentrations of UA, from 5 μM to 0.2 mM. Scan rate of 10 mV·s^−1^. Inset: plot of peak currents vs. concentration of UA.

We tested the suitability of HMT-PMBI-coated electrodes for the detection of UA in a sample of artificial urine, Surine^®^. Figure 9 shows the DPVs of Surine^®^ obtained by bare GCE (Figure 9a) and 1% HMT-PMBI-coated electrode (Figure 9b) at increased concentrations of UA. In the case of the bare GCE, an irreversible oxidation peak occurs at ca. 0.37 V, whereas in the case of the modified electrode the peak current is slightly higher than bare GCE and occurring at ca. 0.475 V, i.e., it shifts 0.105 V towards a more positive potential. This behavior is typical of coated electrodes in which the redox species incorporated within the polymer films leads to higher peak currents and shifts to more positive potentials [22].

Similar to previous experiments, we performed a DPV study on Surine^®^ recorded at a constant concentration of UA and adding different concentrations of AA (see Figure S10). For bare GCE (see Figure S10a), the peaks related to AA and UA are not distinguishable and only an oxidation peak at ca. 0.33 V is visible, whereas for the 1% HMT-PMBI the oxidation peak of UA occurs at 0.42 V, while a peak corresponding to AA appears at ca. 0.3 V, which increases with the AA concentration. Even though the peak separation between AA and UA is more pronounced, the concomitant detection of both species is problematic. In fact, at higher concentrations of AA, only a broad peak that includes both AA and UA is noticeable. Based on previous experiments, we ran chronoamperometric experiments (see Figure S11) for both the bare GCE and 1% HMT-PMBI-coated electrodes at different concentrations of UA. The current increased linearly with the concentrations of UA and the calculated LoD was 55 μM; however, with DPV the calculated LoD was 6.5 μM. Even though these values are not the best reported in the literature (see Table 2), the simplicity of the material investigated here (consisting of an ionene coating only) and the fact that the samples do not require any treatment are advantageous for the analysis of real samples. It is evident from the experiments that AA interferes with the detection of UA since AA is oxidized at lower potentials. To overcome this issue, the general recommendation is to record CAs at different potentials, e.g., 0.3 V and 0.6 V. In the former case, a calibration curve will be formed with respect to AA, which will allow us to establish the concentration of AA in the real sample. However, in the latter, a calibration curve obtained at 0.6 V with UA will allow us to calculate the concentration of UA after proper subtraction of the concentration obtained at 0.3 V for AA.

**Figure 10:**
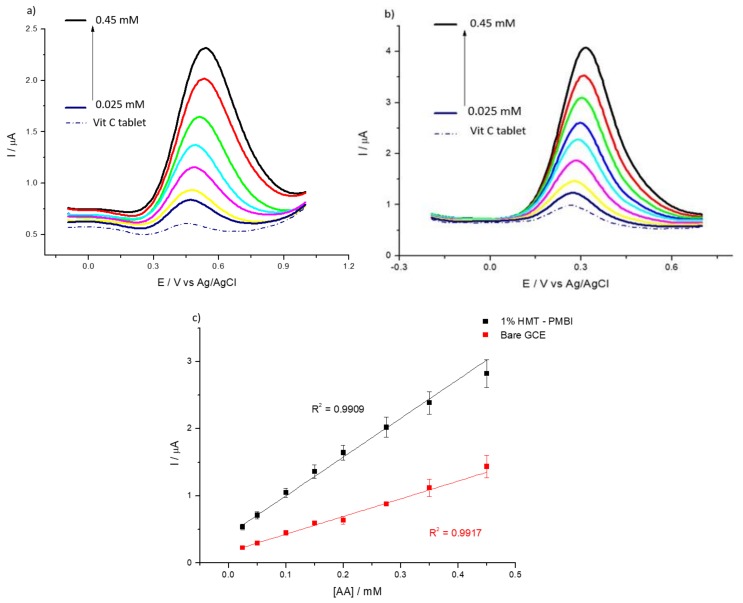
DPVs of bare GCE (**a**) and of 1% HMT-PMBI-coated electrode (**b**) recorded in Vitamin C tablet in the presence of various concentrations of AA, from 25 μM to 450 mM. Scan rate of 10 mV·s^−1^. Plot of peak current vs. concentration of AA (**c**); bare GCE (red line), HMT-PMBI-coated electrode (black line).

Finally, HMT-PMBI-coated electrodes were tested for the detection of AA in Vitamin C tablets. Figure 10 depicts the DPVs for the bare GCE (Figure 10a) and the 1% HMT-PMBI obtained using a Vitamin C tablet and after the addition of AA. In the case of the bare GCE, the oxidation peak of AA occurs at ca. 0.42 V, whereas for the ionene-coated electrode the oxidation peak is facilitated since it occurs at ca. 0.25 V.

The plot of the peak current vs. the concentration of AA shows the enhanced sensitivity of the coated electrode compared to the bare GCE. The calculated sensitivity of HMT-PMBI is 0.082 µA·µM^−1^·cm^−2^ with the LoD and LoQ calculated as 41 µM and 125 µM, respectively, and a linear range between 25 μM–450 μM. The results showed good reproducibility and the RSD values for the ascorbic acid spiked in Vitamin C are less than 5% (see Table 3). The electrochemical investigation also pointed to the good long-term stability of the HMT-PMBI-coated electrodes. In fact, the same HMT-PMBI-coated electrode can be used for up to a week without significant losses (<5%) in the voltammetric signal (data not shown). All the results show that the as-prepared HMT-PMBI-coated electrode is a simple but competitive platform compared to other materials utilized in electroanalysis for the determination of negatively charged analytes.

## 4. Conclusions

We have reported for the first time the basic electrochemical properties of HMT-PMBI-coated films and studied the possibility of using such a coating in voltammetric detection for sensing applications. The electrochemical characterization was performed using negatively charged redox mediators such as Fe(CN)_6_^4−/3−^ and IrCl_6_^2−/3−^, which allowed for the determination of the mediator concentration within HMT-PMBI-coated films as well as the estimation of the apparent diffusion coefficient. The values of *C_p_* and *D_app_* were found to be in agreement with those reported in the literature for other positively charged ionomers. HMT-PMBI-coated films showed permselectivity properties and were effective at rejecting positively charged redox mediators and dopamine. HMT-PMBI-coated electrodes were tested for the determination of uric acid in artificial urine, Surine^®^ and ascorbic acid in Vitamin C tablets. The results indicated that the as-prepared HMT-PMBI-coated films can detect uric acid with a limit of detection (LoD) of 7.7 μM, a limit of quantification (LoQ) of 23.3 µM, a sensitivity of 1.4 µA·µM^−1^·cm^−2^, and a linearity range between 5 and 200 μM; and ascorbic acid with a limit of detection (LoD) of 41.4 μM, a limit of quantification (LoQ) of 125.5 µM, a sensitivity of 1.4 µA·µM^−1^·cm^−2^, and a linearity range between 25 and 450 μM. The results reported here suggest that a HMT-PMBI-modified electrode can be an effective material for the amperometric detection of redox active anionic species.

## Figures and Tables

**Table 1:** Parameters obtained using cyclic voltammetry and chronocoulometry relating to 0.5%, 0.75% and 1% HMT-PMBI-coated electrodes loaded in 5 mM K_4_Fe(CN)_6_ and 3 mM K_2_IrCl_6_ after transferring to 0.1 M NaCl supporting electrolyte.; S = Anson slope of I_p_ vs. t^1/2^, Φ = film thickness, C_P_ = concentration of K_4_Fe(CN)_6_ and K_2_IrCl_6_ in HMT-PMBI, m = number of moles of K_4_Fe(CN)_6_ and K_2_IrCl_6_ incorporated into the film.

[HMT-PMBI] %	Φ/(10^−4^) cm	Γ/(10^−9^) mol cm^−2^	C_p_/(10^−2^) mol dm^−3^	m/(10^−10^) moles	D_app_/RS(10^−10^) cm^2^s^−1^	S/(10^−4^) Cs^−1/2^	D_app_/Anson(10^−10^) Cm^2^s^−1^
**K_4_Fe(CN)_6_**							
**0.5**	0.49 ± 0.03	1.95 ± 0.40	3.92 ± 0.79	1.39 ± 0.28	**2.51 ± 0.74**	1.25 ± 0.08	**2.61 ± 0.42**
**0.75**	0.69 ± 0.21	8.50 ± 0.72	10.38 ± 1.04	6.03 ± 0.51	**4.72 ± 0.91**	1.11 ± 0.07	**1.12 ± 0.13**
**1**	1.09 ± 0.13	10.15 ± 0.80	9.35 ± 0.74	7.21 ± 0.57	**6.44 ± 1.06**	1.21 ± 0.07	**2.42 ± 0.61**
**K_2_IrCl_6_**					**D_app_/RS(10^−9^) cm^2^s^−1^**		**D_app_/Anson(10^−9^) Cm^2^s^−1^**
**0.5**	0.49 ± 0.03	3.08 ± 0.48	6.19 ± 0.96	2.18 ± 0.34	**1.53 ± 0.53**	2.19 ± 0.14	**1.11 ± 0.38**
**0.75**	0.69 ± 0.21	3.04 ± 0.27	4.40 ± 0.39	2.16 ± 0.19	**3.71 ± 0.72**	1.74 ± 0.02	**1.28 ± 0.26**
**1**	1.09 ± 0.13	6.23 ± 1.14	5.74 ± 1.05	4.43 ± 0.81	**2.72 ± 0.85**	1.65 ± 0.08	**3.11 ± 1.24**

**Table 2:** Analytical performances of amperometric sensors for the detection of uric and ascorbic acid.

Electrode	Linear Range UA (µM)	LoD UA (µM)	Linear Range AA (µM)	LoD AA (µM)	Technique	Reference
MWCNT/poly VA GCE	5–120	1.5	5–120	3.5	CV	[69]
PANi/rGO GCE	2–18	0.2	25–200	20	DPV	[70]
CTAB/rGO/ZnS/GCE	1–500	0.4	1–500	30	DPV	[71]
GO-PAN GCE	3–26	1	150–1050	50	DPV	[72]
HNP-PtTi	100–1000	5.3	200–1000	24.2	DPV	[73]
rGO/AuNPs GCE	0.1–100	2.2	0.01–1	5.7	DPV	[74]
PdAu@RGO/GCE	2.5–66.25	2.5	12.5–700	12.5	DPV	[75]
HMT-PMBI GCE in NaCl	5–200	25	20–750	33	DPV	This work
HMT-PMBI GCE in Surine^®^	5–200	7.7	-	-	DPV	This work
HMT-PMBI GCE in Vit. C tablet	-	-	25–450	41	DPV	This work

**Table 3:** Analytical performance of HMT-PMBI-coated electrode for the detection of ascorbic acid in a commercial vitamin C tablet (S/N = 3)**.**

Sample	Added (µM)	Found (µM)	Recovery %
Vitamin C	-	50 ± 4.39	-
	100	147.93 ± 6.41	98.6%

## Supplementary Materials

The following are available online at https://www.mdpi.com/1424-8220/20/2/443/s1, Figure S1: CVs of 1% HMT-PMBI-coated film recorded during loading in an aqueous solution of 5 mM K_4_Fe(CN)_6_ (a) and 3 mM K_2_IrCl_6_ (b); supporting electrolyte 0.1 M NaCl. Scan rate of 100 mV·s^−1^, Figure S2: Theory and equations based on the calculations of diffusion coefficients for both methods (Randles‒Sevcik and Anson), Figure S3: CVs of 1% HMT-PMBI-coated film loaded in 5 mM K_4_Fe(CN)_6_ (a) and 3 mM K_2_IrCl_6_ (b) after transferring to 0.1 M NaCl supporting electrolyte and continuous cycling for 1 h. Scan rate: from 100 mV·s^−1^, Figure S4: CVs of 1% HMT-PMBI-coated electrode recorded in a solution containing 1mM UA at pH 7 (a) and 1 mM AA at pH 4 (b) (black) and immediately after transferring to 0.1 M NaCl supporting electrolyte (red). Scan rate of 50 mV·s^−1^, Figure S5: CVs of 1% HMT-PMBI-coated electrode recorded in 1mM UA at pH 7 (a) and 1 mM AA at pH 4 (b) at different loading times; supporting electrolyte: 0.1 M NaCl; Scan rate of 50 mV·s^−1^, Figure S6: CVs of 1% HMT-PMBI-coated electrodes recorded in 1 mM UA (a) and 1 mM AA (b) at different pH values; supporting electrolyte: 0.1 M NaCl. Scan rate of 50 mV·s^−1^, Figure S7: DPVs of bare GCE (a) and of 1% HMT-PMBI-coated electrode (b) recorded in 0.1M NaCl in the presence of constant 0.05 mM AA and various concentrations of DA, from 75 μM to 1 mM for (a) and 0.2 mM to 2 mM for (b). Scan rate of 10 mV·s^−1^. Inset: plot of peak currents vs. concentration of DA, Figure S8: Chronoamperometric (*i-t*) response of bare (a) and of 1% HMT-PMBI-coated electrode (b) obtained with successive concentration of AA from 5 μM to 0.2 mM recorded in 0.1 M NaCl supporting electrolyte (pH 4), applied potential 0.3 V. (c) Calibration plot as a function of AA concentration as in (a,b). Error bars calculated from three repeat measurements, Figure S9: Chronoamperometric (*i-t*) response of bare (a) and 1% HMT-PMBI-coated electrode (b) obtained with successive concentration of UA from 5 μM to 0.3 mM recorded in 0.1 M NaCl supporting electrolyte (pH 7); applied potential 0.6 V. (c) Calibration plot as a function of UA concentration as in (a,b). Error bars calculated from three repeat measurements, Figure S10: DPVs of bare GCE (a) and 1% HMT-PMBI-coated electrode (b) recorded in Surine^®^ in the presence of constant 0.05 mM UA and various concentrations of AA, from 0.05 mM to 1 mM for (a) and 0.05 mM to 2 mM for (b). Scan rate of 10 mV·s^−1^. Inset: plot of peak currents vs. concentration of UA, Figure S11: Chronoamperometric (*i-t*) response of bare GCE (a) and 1% HMT-PMBI-coated GCE (b) applying 0.6 V obtained with successive concentration of UA from 5 μM to 0.75 mM of (a) and 5 μM–1 mM for (b) in Surine^®^ (pH 6.8).
