# Supplementary material for: Voltammetry at Hexamethyl-P-Terphenyl Poly(Benzimidazolium) (HMT-PMBI)-Coated Glassy Carbon Electrodes: Charge Transport Properties and Detection of Uric and Ascorbic Acid
Source: Sensors (Basel). 2020 Jan 13;20(2):443. doi: 10.3390/s20020443 (PMC7013716; doi:10.3390/s20020443)
Supplement: Supplementary file 1 [file sensors-20-00443-s001.pdf]

sensors

Electronic Supporting Information (ESI)

Voltammetry at Hexamethyl-p-terphenyl poly(benzimidazolium) (HMT-PMBI)-Coated Glassy Carbon Electrodes: Charge Transport Properties and Investigation towards Detection of Uric and Ascorbic Acid

Matthew Rees [1], Andrew G. Wright [2], Steven Holdcroft [2] and Paolo Bertoncello [1,3,*]

S1.

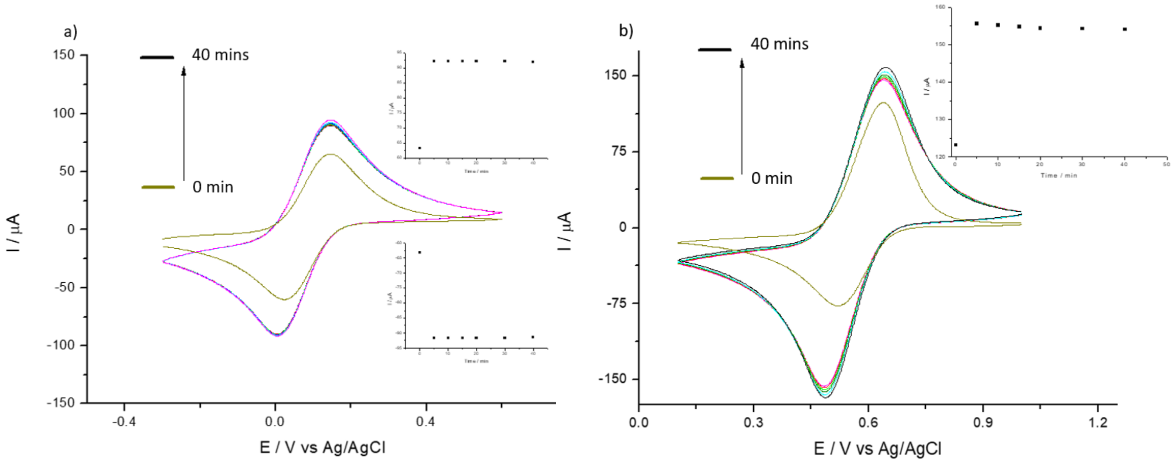

Figure 1. CVs of 1% HMT-PMBI coated film recorded during loading in an aqueous solution of 5 mM $K_4Fe(CN)_6$ (**a**) and 3 mM K_2IrCl_6 (**b**); supporting electrolyte 0.1 M NaCl. Scan rate of 100 mV s^{-1}.

S2.

The surface coverage values (Γ, mol cm^{-2}) of HMT-PMBI coated films with incorporated the redox probes $K_4Fe(CN)_6$ or K_2IrCl_6 were calculated from the CVs recorded at low scan rates, after transferring the modified electrodes into the supporting electrolyte solution without the redox specie (Figure 1a,c), e.g., when the CVs display thin-layer characteristics using the relation:

$$\Gamma = \frac{Q}{nFA} \quad (1)$$

where Q (C) is the charge on the forward or reverse scan, n is the number of electrons transferred (n = 1 for both $K_4Fe(CN)_6$ and K_2IrCl_6), A (cm^2) is the geometric area of the electrode, and F is the Faraday constant (96486 C mol^{-1}). These values can also be expressed in terms of concentration, C_0^*, by dividing the surface coverage with the thickness of HMT-PMBI coated film, Φ (cm), calculated in dry conditions using the profilometer, e.g.,

$$C_0^* = \frac{\Gamma}{\Phi} \quad (2)$$

The apparent diffusion coefficients were calculated by using two methods, e.g., (1) the Randles-Sevcik and (2) the Anson's plot methods. For the method using the Randles-Sevcik equation[1], e.g.,

$$I_{p\,a,c} = (2.69x10^5)n^{3/2}AD_{app}^{1/2}C_0^*v^{1/2} \quad (3)$$

we plotted the anodic peak currents, $I_{p,a}$ in the case of $Fe(CN)_6^{2-}$ or the cathodic peak current $I_{p,c}$ in the case of $IrCl_6^{2-}$ versus the square root of the scan rate, $v^{1/2}$, with the tacit assumption that the redox process is reversible. To underline the fact that the ΔE_P for each recorded CV increased monotonically with the scan rate and this will lead to an underestimation of D_{app}. The slope of these plots recorded in the faster scan rate regime, combined with thickness of the polymer film evaluated using the profilometer and the number of electroactive species obtained by coulometric integration of the anodic (or cathodic) peak currents under thin-layer conditions allowed the evaluation of the apparent diffusion coefficient values of HMT-PMBI coated electrodes. Instead, for the Anson's method, potential-step chronocoulometry was used to determine the values of D_{app} from the slope of the plots of the charge Q vs. the square root of time, $t^{1/2}$, using the relation[2]:

$$D_{app} = \left[\frac{S\Phi\pi^{1/2}}{2F\Gamma}\right]^2 = \left[\frac{S\pi^{1/2}}{2FC}\right]^2 \quad (4)$$

where S is the chronocoulometric slope (C cm^{-2} t$^{-1/2}$), and Γ, Φ, and F with the conventional meaning as previously mentioned.

S3.

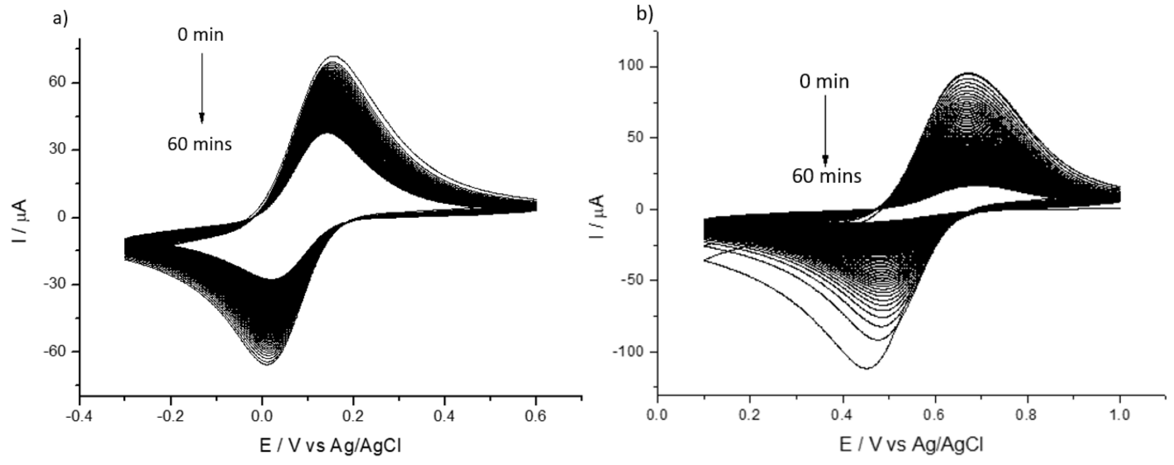

Figure 3. CVs of 1% HMT-PMBI coated film loaded in 5 mM $K_4Fe(CN)_6$ (**a**) and 3 mM K_2IrCl_6 (**b**) after transferring to 0.1 M NaCl supporting electrolyte and continuous cycling for 1 hour. Scan rate: from 100 mV s^{-1}.

S4.

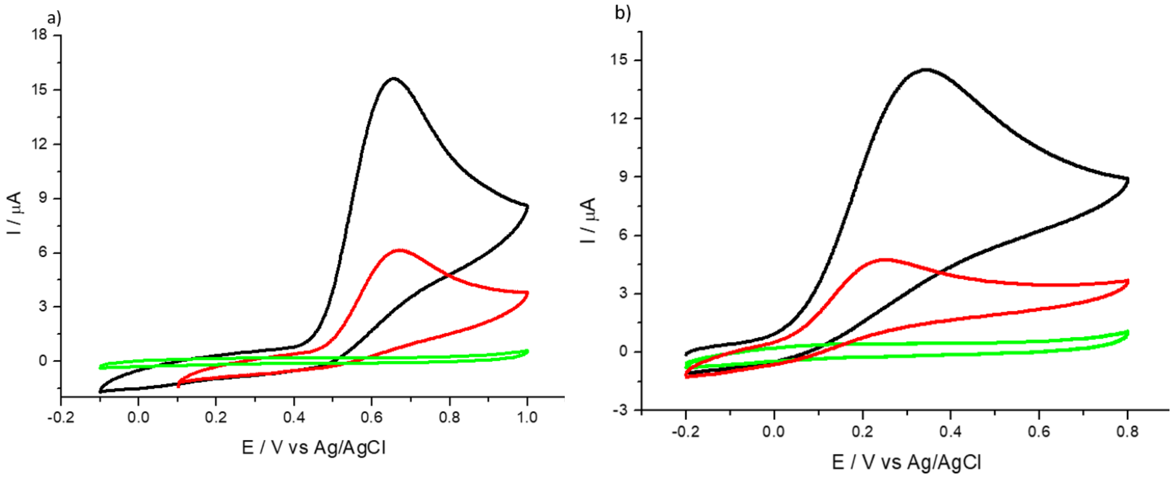

Figure 4. CVs of 1% HMT-PMBI coated electrode recorded in a solution containing 1mM UA at pH 7 (**a**) and 1 mM AA at pH 4 (**b**) (black) and immediately after transferring in 0.1 M NaCl supporting electrolyte (red); Scan rate of 50 mV s^{-1}.

S5.

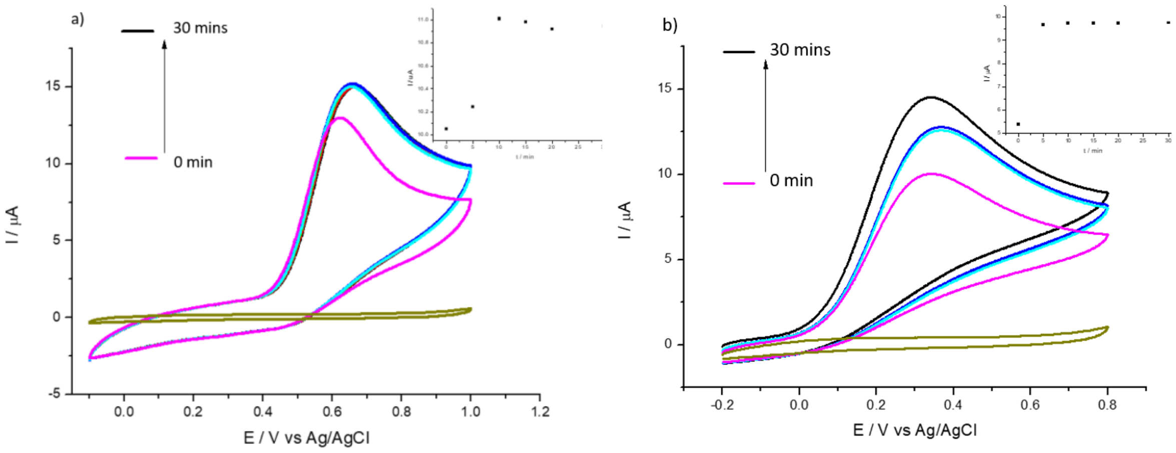

Figure 5. CVs of 1% HMT-PMBI coated electrode recorded in 1mM UA at pH 7 (**a**) and 1 mM AA at pH 4 (**b**) at different loading time; supporting electrolyte: 0.1 M NaCl; Scan rate of 50 mV s^{-1}.

S6.

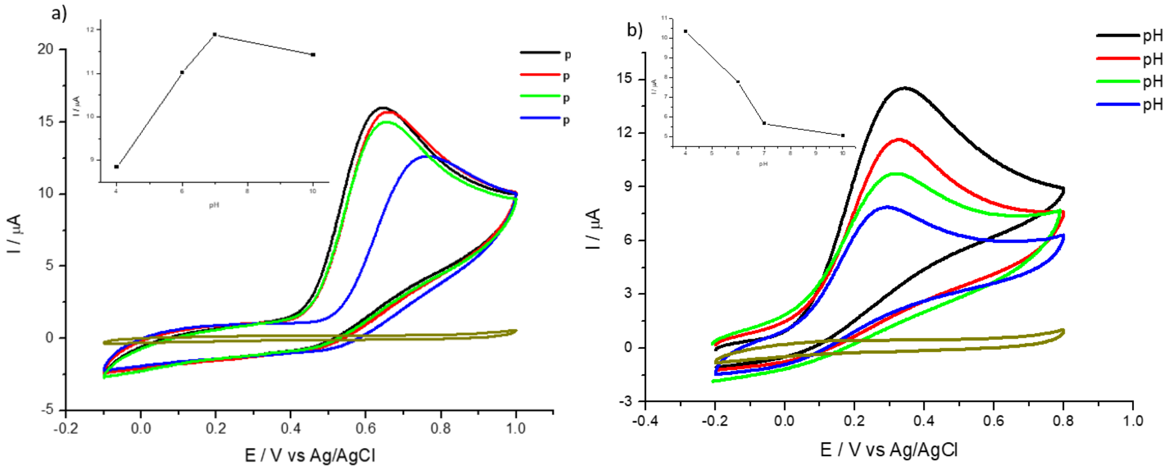

Figure 6. CVs of 1% HMT-PMBI coated electrodes recorded in 1 mM UA (a) and 1 mM AA (b) at different pH values; supporting electrolyte: 0.1 M NaCl; Scan rate of 50 mV s^{-1}.

S7.

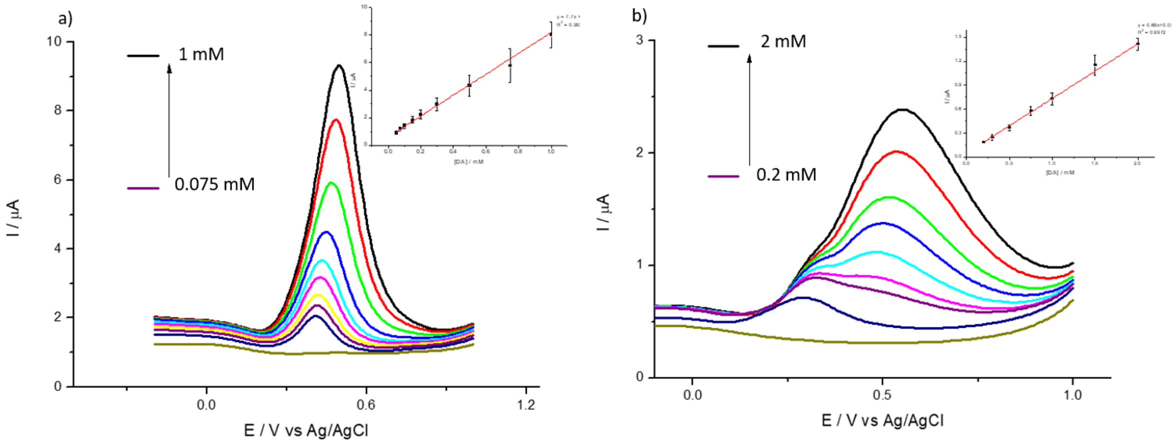

Figure 7. DPVs of bare GCE (**a**) and of 1% HMT-PMBI (**b**) coated electrode recorded in 0.1M NaCl in the presence of constant 0.05 mM AA and various concentrations of DA, from 75 µM to 1 mM for (**a**) and 0.2 mM to 2 mM for (**b**). Scan rate of 10 mV s^{-1}. Inset: plot of peak currents *vs.* concentration of DA.

S8.

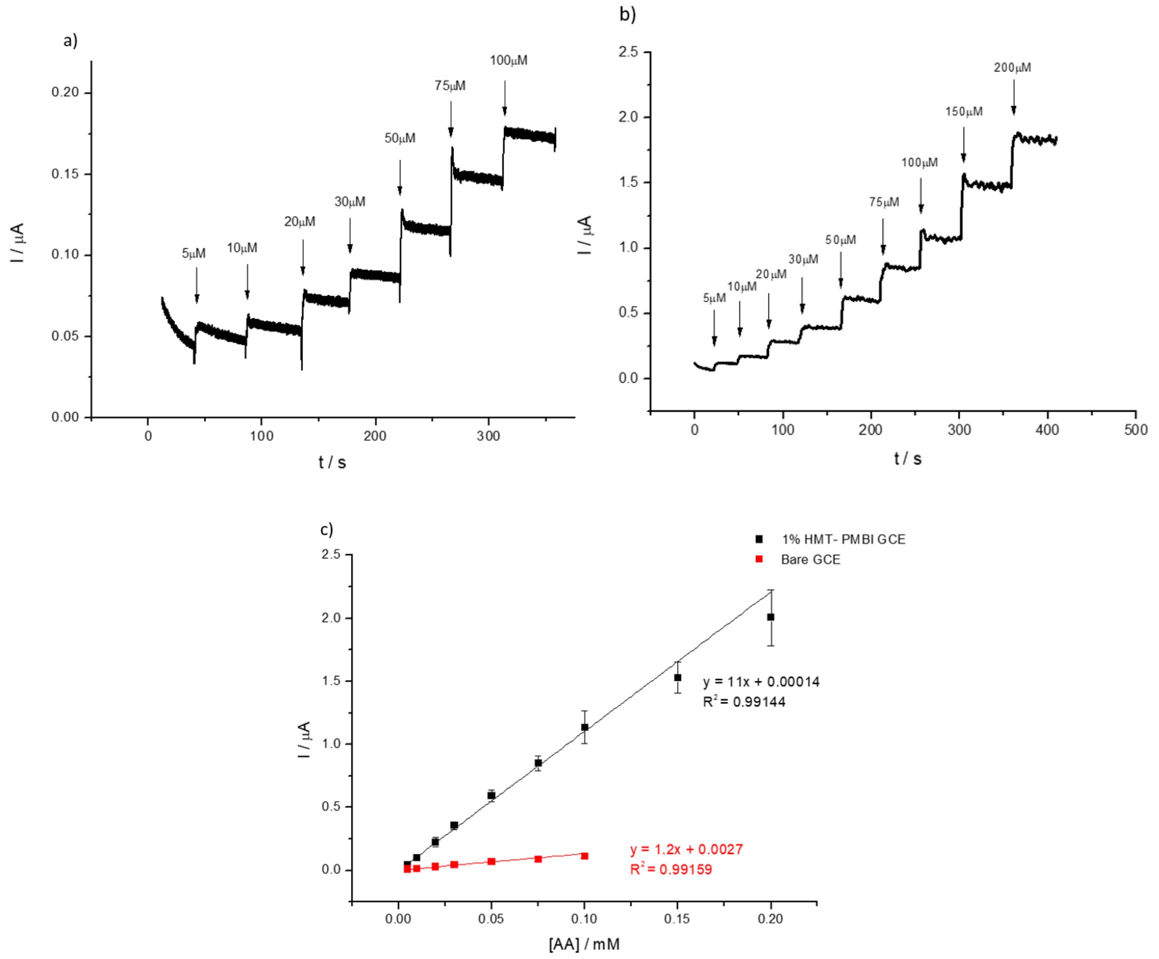

Figure 8. Chronoamperometric (*i-t*) response of bare (**a**) and of 1% HMT-PMBI (b) coated electrode obtained with successive concentration of AA from 5 µM to 0.2 mM recorded in 0.1 M NaCl supporting electrolyte (pH 4), applied potential 0.3 V. (**c**) Calibration plot as a function of AA concentration as in (**a**, **b**). Error bars calculated from 3 repeat measurements.

S9.

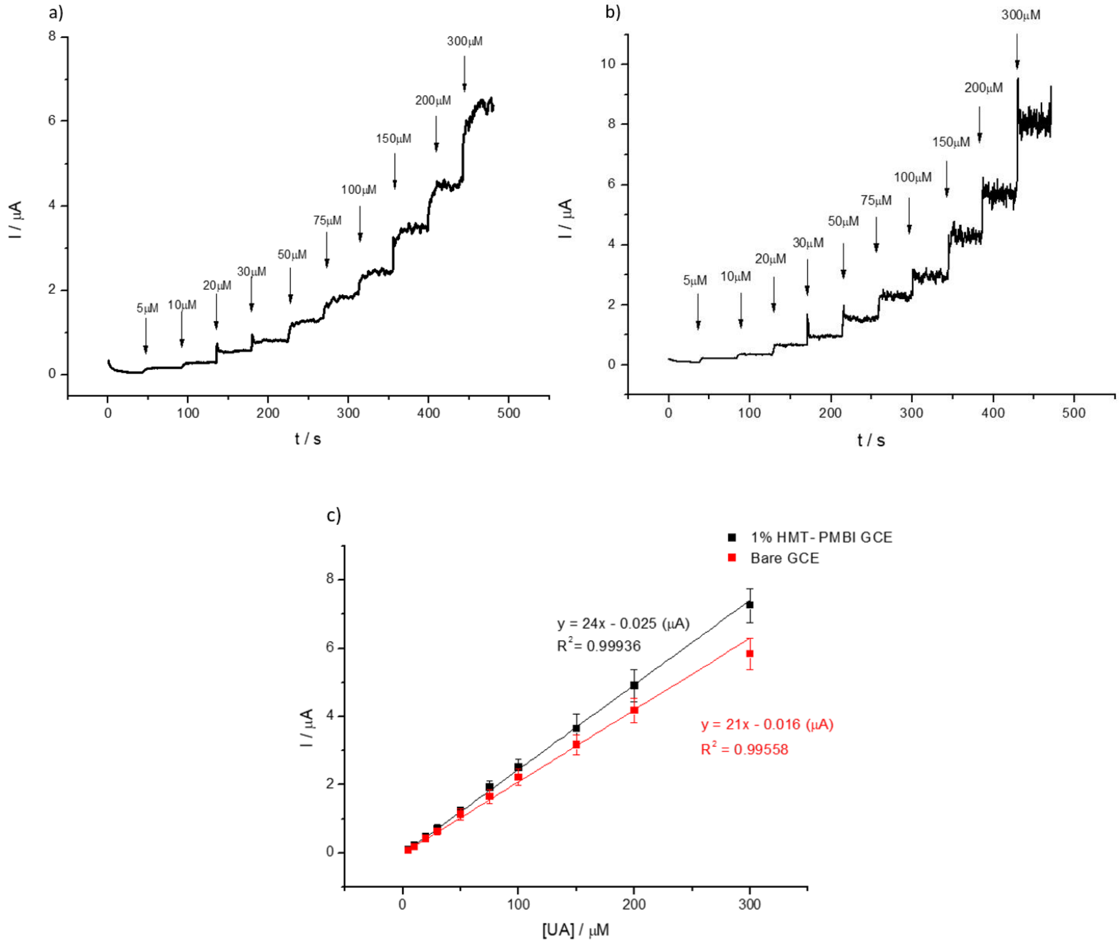

Figure 9. Chronoamperometric (*i*-*t*) response of bare (**a**) and of 1% HMT-PMBI (**b**) coated electrode obtained with successive concentration of UA from 5 µM to 0.3 mM recorded in 0.1 M NaCl supporting electrolyte (pH 7), applied potential 0.6 V. (**c**) Calibration plot as a function of UA concentration as in (**a**, **b**). Error bars calculated from 3 repeat measurements.

S10.

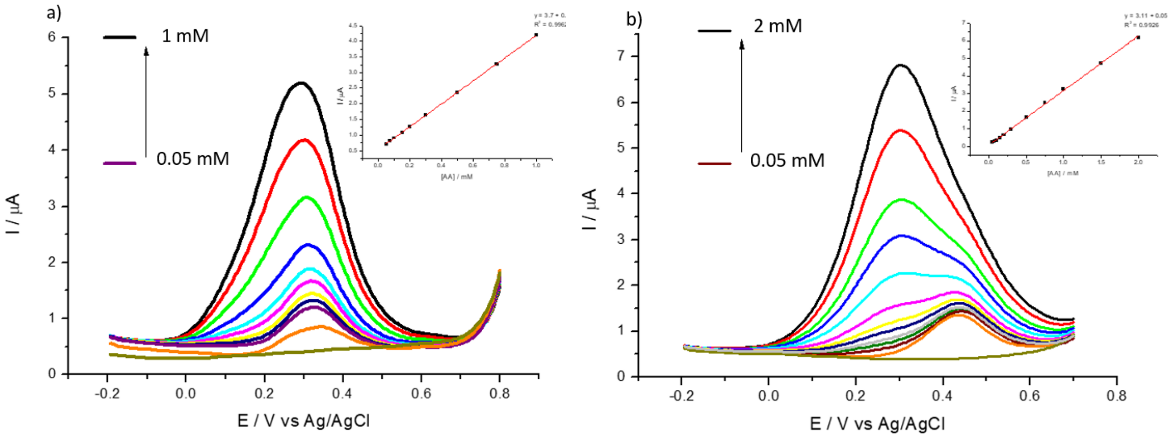

Figure 10. DPVs of bare GCE (**a**) and of 1% HMT-PMBI (**b**) coated electrode recorded in Surine® in the presence of constant 0.05 mM UA and various concentrations of AA, from 0.05 mM to 1 mM for (**a**) and 0.05 mM to 2 mM for (**b**). Scan rate of 10 mV s^{-1}. Inset: plot of peak currents *vs.* concentration of UA.

S11.

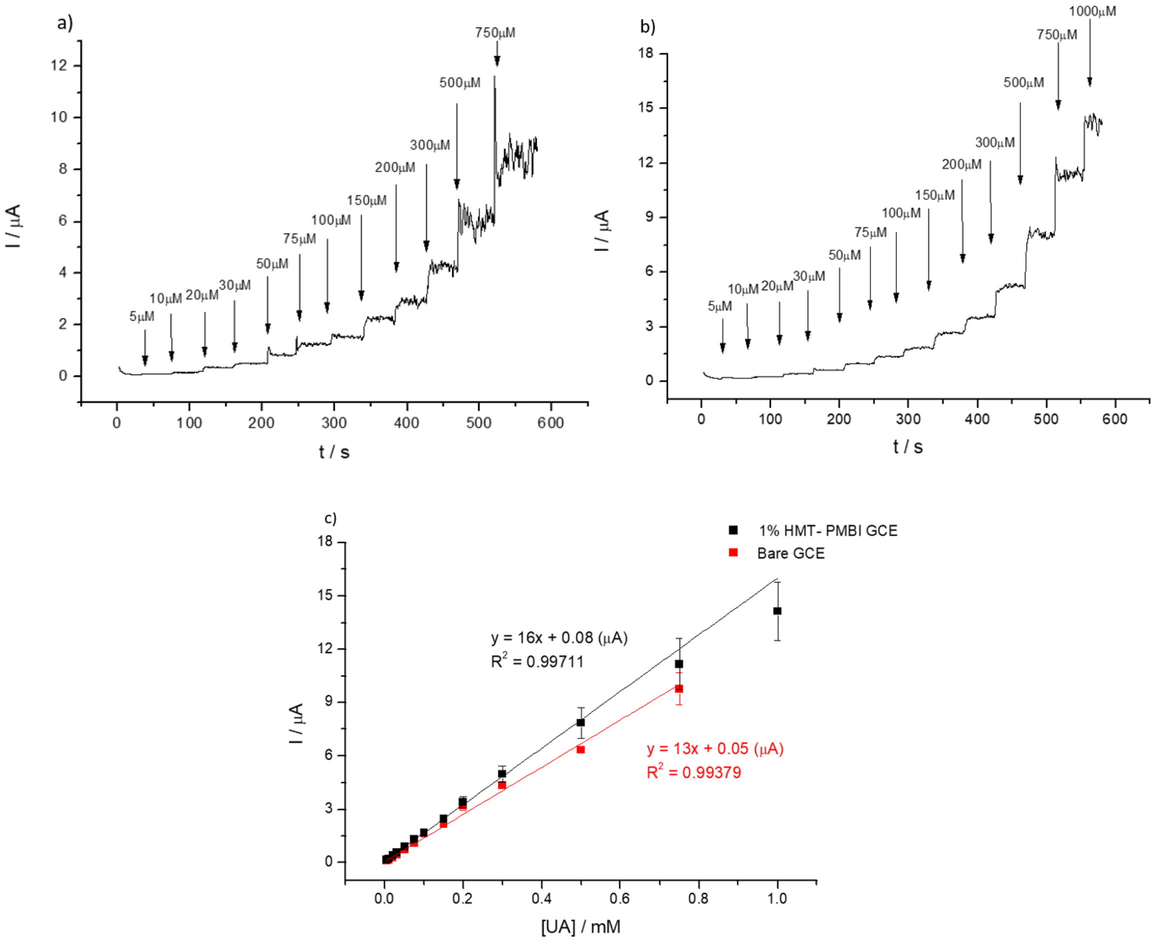

Figure 11. Chronoamperometric (*i*-*t*) response of bare GCE (**a**) and 1% HMT-PMBI (b) coated GCE applying 0.6 V respectively obtained with successive concentration of UA from 5 µM–0.75 mM of (**a**) and 5 µM–1 mM for (**b**) in Surine® (pH 6.8).

References

1. Bard, A.J. and L.R. Faulkner, *Electrochemical Methods: Fundamentals and Applications*. 2001, John Wiley & Sons Inc.: New York. p. 226-260.
2. Buttry, D.A. and F.C. Anson, *EFFECTS OF ELECTRON EXCHANGE AND SINGLE-FILE DIFFUSION ON CHARGE PROPAGATION IN NAFION FILMS CONTAINING REDOX COUPLES.* Journal of the American Chemical Society, 1983. **105**(4): p. 685-689.
